# Rhizodegradation of PAHs differentially altered by C3 and C4 plants

**DOI:** 10.1038/s41598-020-72844-4

**Published:** 2020-09-30

**Authors:** Anithadevi Kenday Sivaram, Suresh Ramraj Subashchandrabose, Panneerselvan Logeshwaran, Robin Lockington, Ravi Naidu, Mallavarapu Megharaj

**Affiliations:** 1 Global Centre for Environmental Remediation, The University of Newcastle, University Drive, Callaghan, NSW 2308 Australia; 2 Cooperative Research Centre for Contamination Assessment and Remediation of Environment, Advanced Technology Centre, The University of Newcastle, University Drive, Callaghan, NSW 2308 Australia; 3 Centre for Environmental Risk Assessment and Remediation, University of South Australia, Adelaide, SA Australia

**Keywords:** Plant sciences, Environmental impact, Microbiology, Environmental microbiology

## Abstract

Pyrosequencing of 16S ribosomal RNA (rRNA) was employed to characterize bacterial communities colonizing the rhizosphere of plants with C3 and C4 photosynthetic pathways grown in soil contaminated with polycyclic aromatic hydrocarbons (PAHs) after 60 and 120 days. The results of this study exhibited a clear difference in bacterial diversity between the rhizosphere and non-rhizosphere samples and between the rhizospheres of the C3 and C4 plants after 120 days. In both C3 and C4 rhizospheres, an incremental change in PAHs degrading bacterial genera was observed in the 120th day samples compared to the 60th day ones. Among the PAHs degrading bacterial genera, *Pseudomonas* showed good resistance to PAHs in the 120th day rhizosphere of both C3 and C4 plants. Conversely, the genus *Sphingomonas* showed sensitivity to PAHs in the 120th day rhizosphere soils of C3 plants only. Also, a significant increase in the PAHs degrading genera was observed at 120th day in the C4 rhizosphere in comparison to the C3 rhizosphere, which was reflected in a reduced PAHs concentration measured in the soil remediated with C4 plants rather than C3 plants. These results suggest that the rhizoremediation of PAHs was primarily governed by the plant photosystems, which led to differences in root secretions that caused the variation in bacterial diversity seen in the rhizospheres. This study is the first report to demonstrate the greater effectiveness of C4 plants in enhancing the PAHs degrading bacterial community than C3 plants.

## Introduction

Polycyclic aromatic hydrocarbons (PAHs) rank top among the persistent, toxic organic pollutants listed by the US Environmental Protection Agency (US EPA)^1^. PAHs are formed naturally through volcanic eruptions, forest fires, or anthropogenic activities such as wood fires, gas production industries, or vehicle exhausts^2,3^. Several remediation strategies involving natural attenuation, thermal, physical, chemical, and biological approaches have been employed to remediate PAHs from the environment. Among them, the effective utilization of plants and their associated microorganisms has been the most common method used to remediate toxic organic pollutants, especially polycyclic aromatic hydrocarbons (PAHs), due to its sustainability, coupled with being cost-effective and environmentally friendly nature^4,5^. Many researchers have reported the phytoremediation of PAHs, both under controlled and field conditions, highlighting the growing evidence for the importance and viability of this technique^6–8^. The plant’s relationship with the microorganisms present in the root zone (rhizosphere) enhances the degradation of PAHs by what is known as the rhizosphere effect, and this involves in the enrichment of PAHs-degrading microorganisms in the rhizosphere by plant root exudates^9^.

Plant roots release a broad range of photosynthetically derived carbon-containing bioactive compounds, collectively known as root exudates or rhizodeposits, into the rhizosphere. These root exudates can perform a variety of functions, including increasing nutrient availability in the root zone^10^, improving plant defense mechanisms, and phytoextraction of heavy metals^11^. In addition to this, the root exudates provide nutrients that lead to increase in the number of microorganisms in the rhizosphere region thereby increasing the rate of microbial degradation of organic pollutants such as PAHs^12,13^. Root exudates also play a vital role in determining the microbial dynamics in the rhizosphere^14^.

Plants are generally divided into three groups, C3, C4, and CAM (Crassulacean Acid Metabolism) based on their photosynthetic carbon fixation pathways. Photosynthesis is a necessary metabolic process that produces sugar for the growth and development of plants^15^. Plants with the C4 photosynthetic pathway can more efficiently convert solar energy to plant biomass than the C3 plants^16^. A significant difference in the compounds such as sugars and organic acids  reported in the root exudates of the C3 and C4 plants has also been reported^17^. The dominant sugars identified in C3 plants were mannose, maltose, and ribose, while inositol, erythritol, and ribitol dominates in the root exudates of C4 plants^18^. Likewise, organic acids such as, fumaric and *trans*-aconitic acid were reported to be dominant in root exudates of only the C4 plants rhizosphere^17^. This difference in the composition of the root exudates between C3 and C4 plants may ultimately lead to the differences in the remediation of organic pollutants, especially PAHs^19,20^.

Changes in the biochemical and physiological responses of plants to environmental factors can also significantly alter root exudates, thus altering the microbial community in the rhizosphere^21^. Moreover, these changes are also believed to be species-specific and vary between plant species and the life stages of the plant, even under similar environmental conditions^22^. Therefore, the root exudates perform a particular function by serving as an active link between the soil, plant, and rhizospheric microbial population. The effect of the root exudates on the rhizosphere’s microbial community involved in the rhizoremediation of organic pollutants remains a subject of debate. Earlier studies reported on the constructive impact of the root exudates in proliferating the microbial population and thus enhancing the degrading population in the rhizosphere selectively^13^. On the other hand, the adverse effect of root exudates was also highlighted, stating that microbial communities were well adapted to low nutrient concentrations in the contaminated soil conditions, and their rate of degradation decreased when provided with excess carbon and energy from the root exudates^23^. A complex mixture of low (LMW) and high molecular weight (HMW) PAHs usually dominates PAHs- contaminated environments. Microbes, especially bacteria, prefer LMW over HMW PAH as their sole source of carbon^24^. Numerous studies have been carried out on PAH degradation by both Gram-positive and Gram-negative bacteria, isolated from the PAHs contaminated soils^25,26^. Among these bacterial isolates, Gram-positive species such as *Mycobacterium* have been most widely reported for their ability to utilize PAHs as sole carbon and energy source^19,27^. Several studies have been published on the potential of microbes to degrade PAHs either by metabolism or co-metabolism^13,19,24^. The process in the metabolism of the low molecular weight PAHs by aerobic bacteria is initiated by the addition of oxygen molecules in the aromatic nucleus by dioxygenase enzymes, which produces dihydrodiols. The dihydrodiols undergo a dehydrogenation process through the action of the enzyme, extradiol dioxygenase. The resulting dehydroxylated intermediates are processed through the *ortho* or a *meta* cleavage pathways forming intermediate products such as protocatechuates and catechols, which undergo a subsequent series of enzymatic actions to form tricarboxylic intermediates^28^. Rhizospheric bacterial genera such as *Bacillus*, *Acinetobacter* sp., *Arthrobacter* sp., *Diaphorobacter* sp., *Enterobacter* sp., *Flavobacterium* sp., *Phanerochaete chrysosporium*, *Polysporus* sp., *Pseudomonas* sp., *Pseudoxanthomonas* sp., *Rhodococcus wratislaviensis*, *Sphingomonas* sp., and *Stenotrophomonas* sp., have all been reported to degrade PAHs such as phenanthrene and pyrene^19,29^.

However, the use of plants alone may not prove to be successful in the remediation process because of the slower growth of plants and their sensitivity to HMW PAHs in the contaminated soils^30^. Therefore, a synergistic relationship between plants and microbes is often required for the effective removal of complex mixtures of PAHs contaminants present in the contaminated environment^31^. A better resolution and greater ability to analyze, envisage, and characterize the diversity of the soil microbial community has been made possible with the Next-generation sequencing techniques^32,33^. The 16 s rRNA based amplicon sequencing technique provides comprehensive information on the differences in structural and functional soil microbial communities in long-term contaminated soils including hydrocarbons polluted soils^34,35^.

In this study, the performance of plants such as cowpea (*Vigna unguiculata*), sunflower (*Helianthus annus*), and wallaby grass (*Austrodanthonia caespitosa*) with C3 photosystems; and maize (*Zea mays*), Sudan grass (*Sorghum sudanense*), and vetiver (*Vetiveria zizanoides*) with C4 photosystems were investigated for their ability to promote root-associated microbiota involved in the rhizodegradation of PAHs contaminated soil (collected from a landfill site in Dublin, South Australia). The C3 and C4 plants were selected based on our previous screening experiment on the degradation of PAHs, physiological characters, and their ability to withstand PAHs^5^. The selected C3 and C4 plants were grown in PAHs contaminated soil (treatments) and unplanted PAHs contaminated soil (control) for 60 and 120 days in three replications in greenhouse conditions. The rhizosphere soil of the selected plants and unplanted control soil were processed for the extraction of soil DNA and sequenced for the 16S rRNA genes using the pyrosequencing technique. This study allowed us to examine the possible impact of the rhizosphere and non-rhizosphere soils, PAHs contamination, plant photosystems (C3 and C4 photosystems), and plant growth stages (60 and 120 days after planting) on the microbial community diversity.

## Results

### Impact of C3 and C4 plants on PAHs degradation

**Figure 1:**
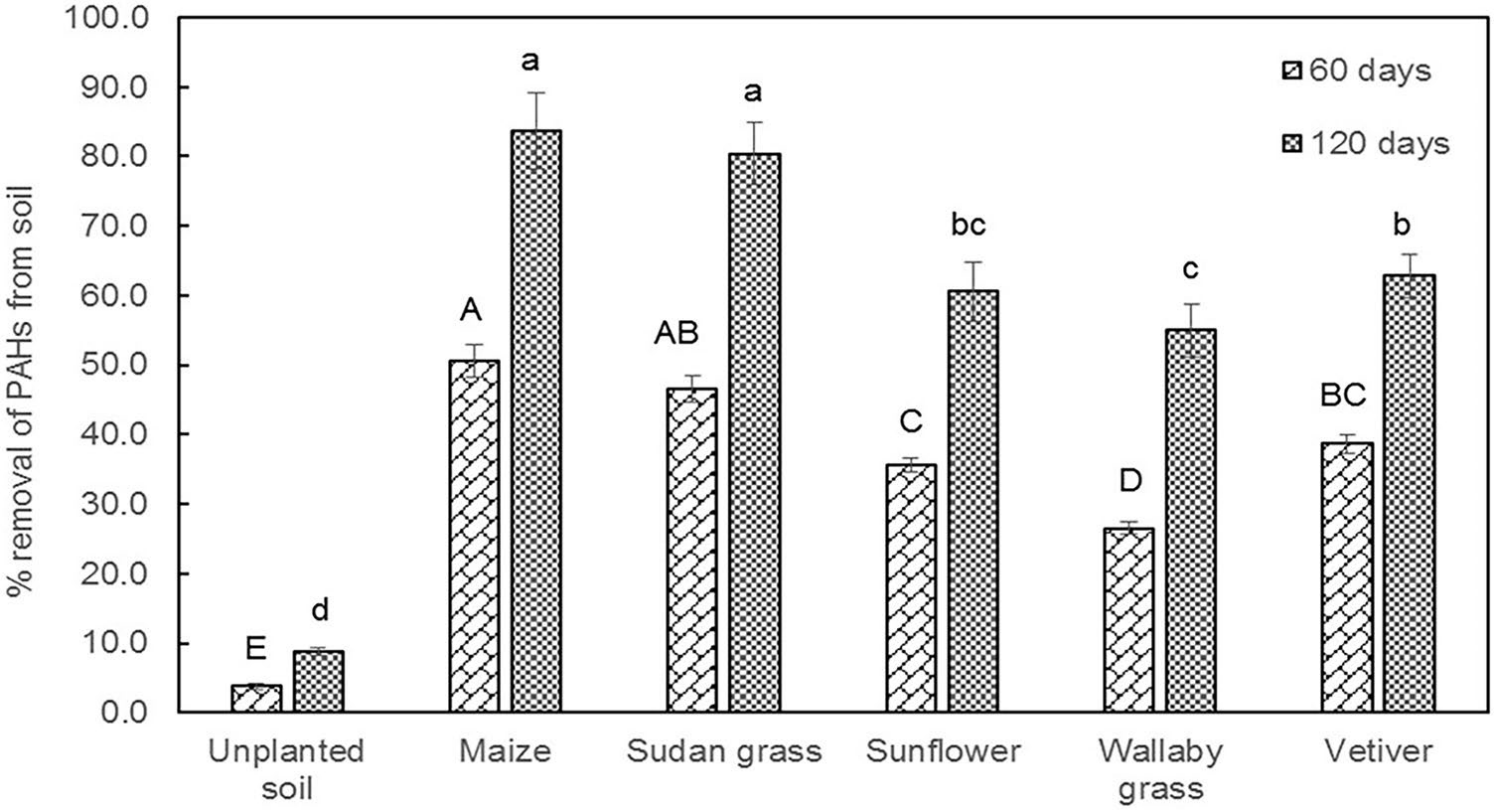
The effect of C3 and C4 plant species in percentage removal of PAHs from the contaminated soil after 60 and 120 days. The data expressed as means and error bar indicates ± SD. Bars with similar letters were statistically not significant at 5% level of probability by DMRT.

The efficacy of C3 and C4 plants in removing PAHs from contaminated soils was examined after the 60th and 120th day of the experimental duration (Fig. 1). Generally, the planted treatments showed enhanced PAHs removal when compared to unplanted PAH contaminated control soils. Interestingly, PAHs removal varied with the plant photosystems (C3 and C4 photosystems). Overall, among the plants, maize and Sudan grass belonging to the C4 group showed higher PAHs removal after the 60 and 120 days than the C3 photosystem group.

### Characterization of rhizosphere microbiota in C3 and C4 plants

**Figure 2:**
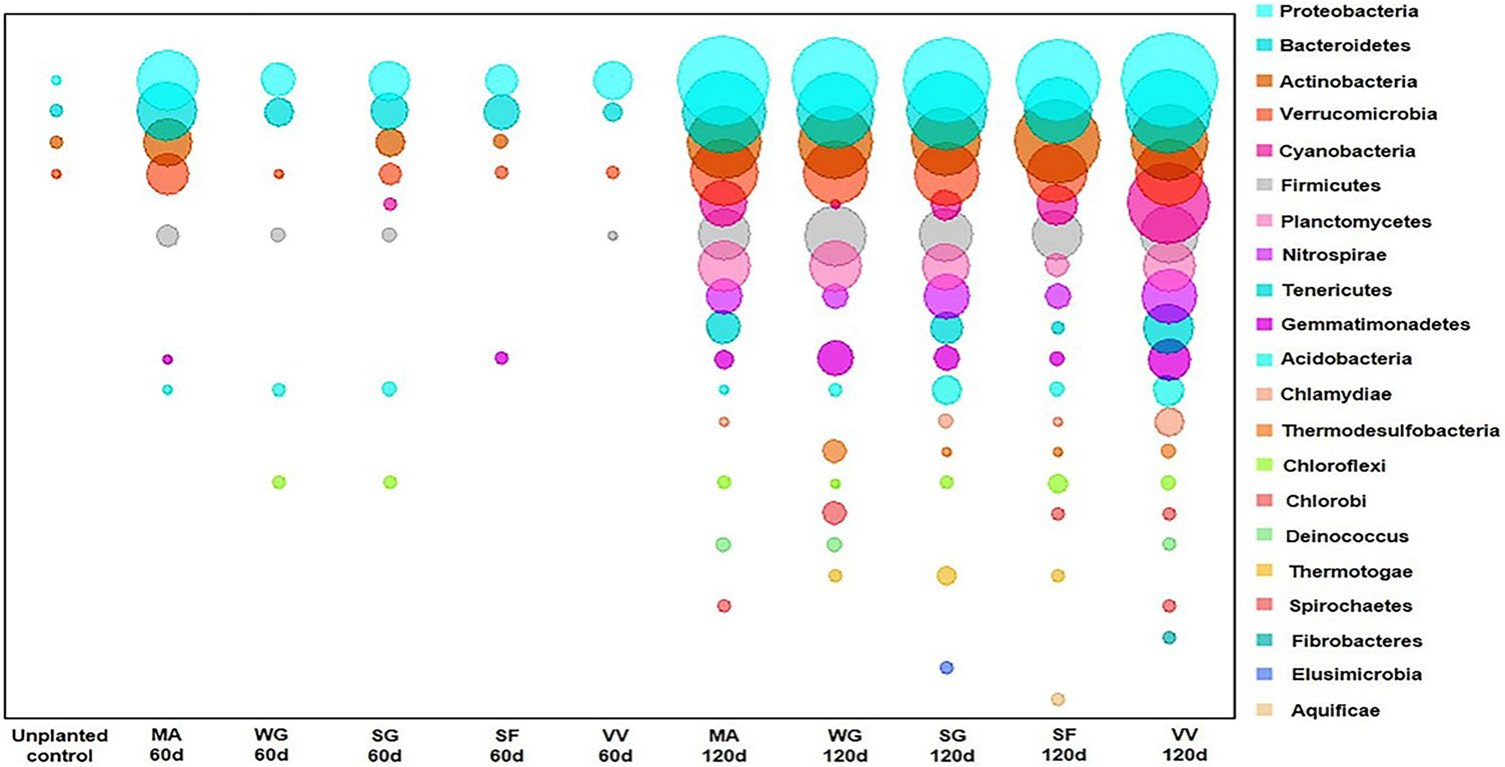
Bacterial phylum-level classification and comparison between planted and unplanted soils. The X-axis represents the number of reads of different bacterial phyla in the log scale. MA—maize, CP—cowpea, SF—sunflower, SG—Sudan grass, VV—vetiver, and WG—wallaby grass.

**Figure 3:**
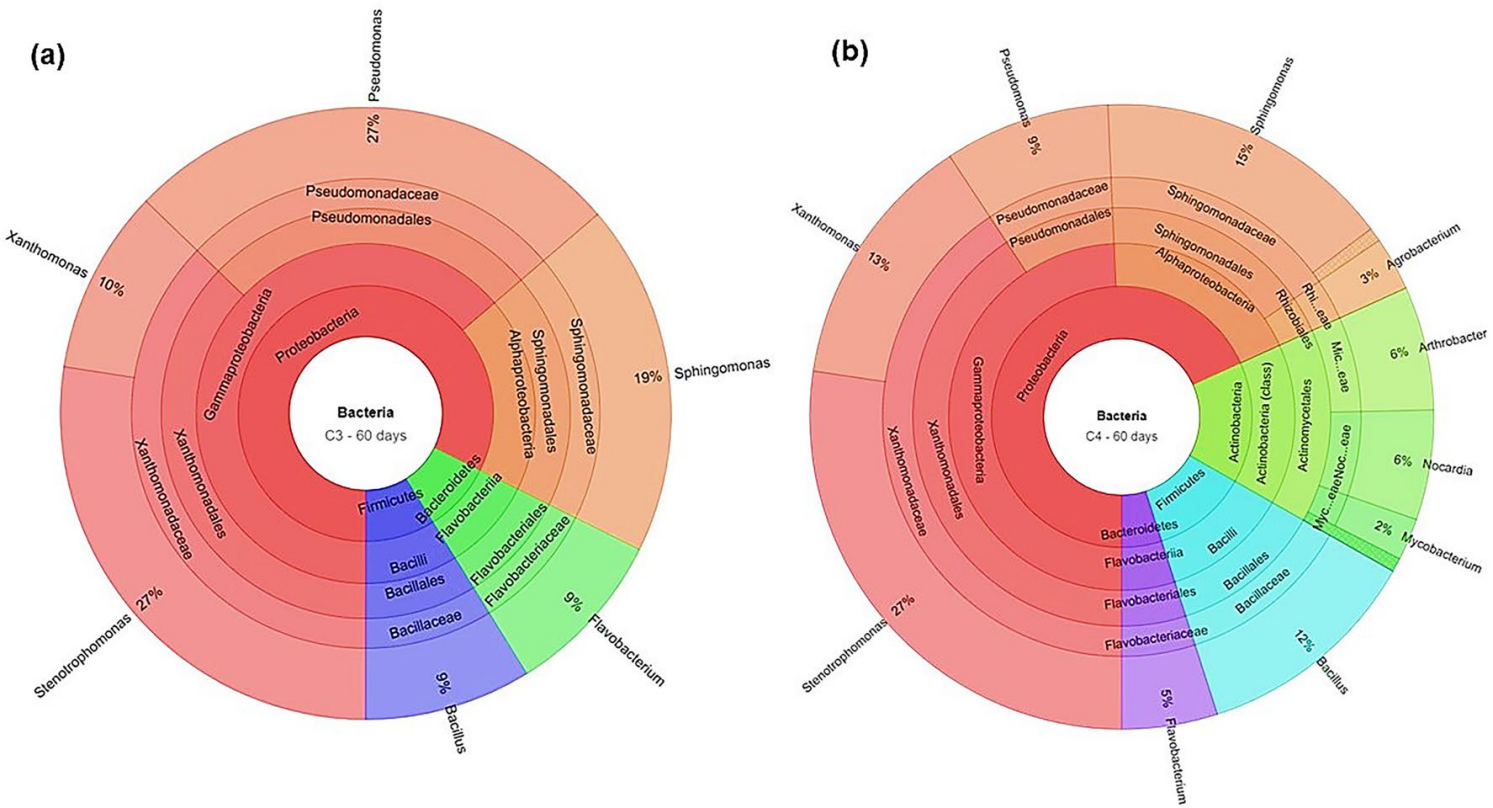
Krona chart highlighting the relative abundance of PAHs degrading bacterial genera after the 60th day in C3 rhizosphere (**a**) and, C4 rhizosphere (**b**).

**Figure 4:**
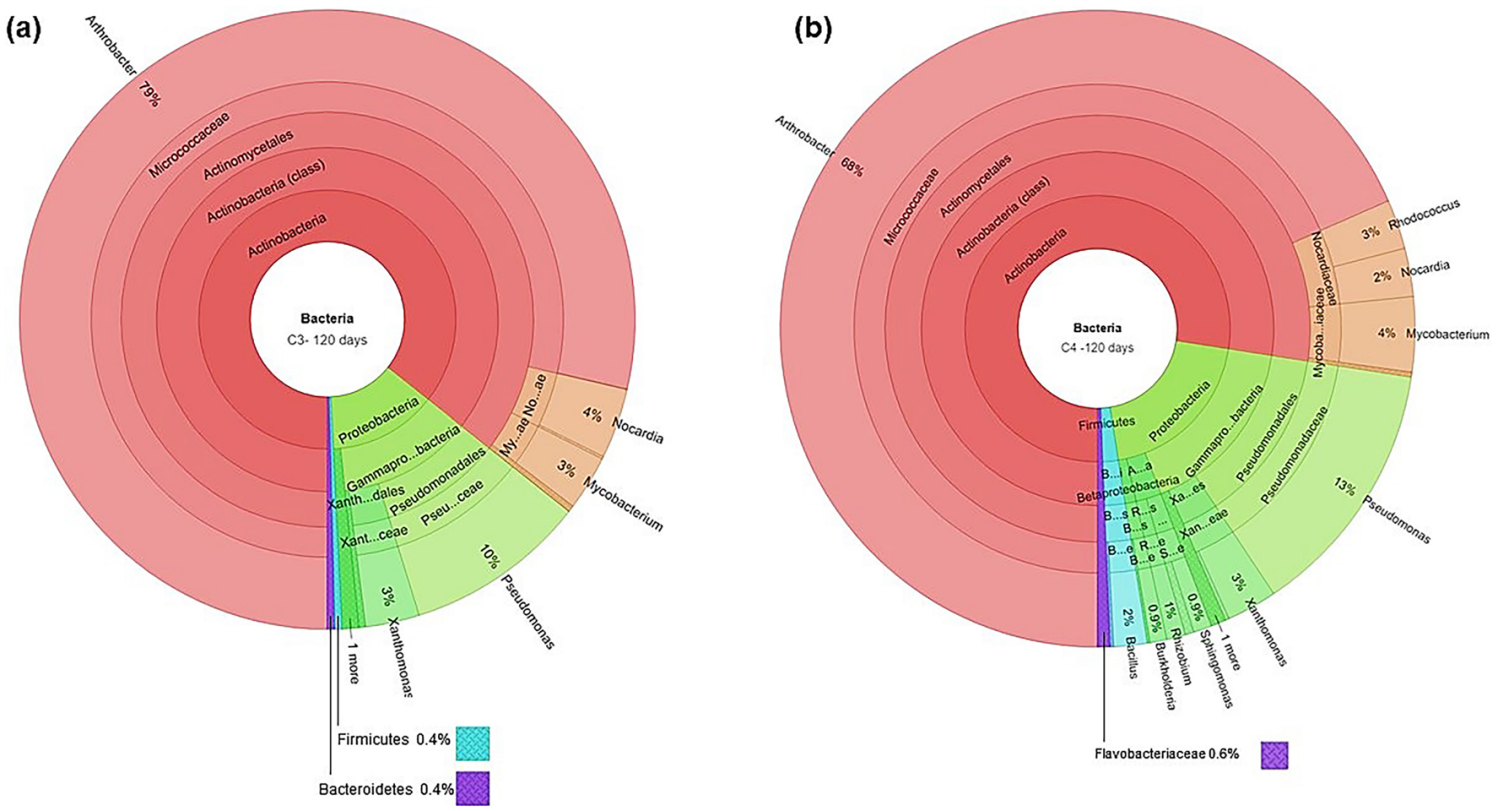
Krona chart highlighting the relative abundance of PAHs degrading bacterial genera after 120th day in C3 rhizosphere (**a**) and, C4 rhizosphere (**b**).

The rhizosphere soil DNA samples from C3 and C4 plants grown in PAHs contaminated soil, and unplanted control soil were collected after 60 and 120 days, for the 16S rRNA pyrosequencing analysis. Since cowpea exhibited poor performance at the end of 60 days, it is excluded from the C3 group for 120th day experiments. Based on the 16S rRNA gene sequence analysis, there was a clear evidence of the extensive variation in the diversity of microbial communities in the rhizosphere of C3 and C4 plants, and unplanted control soil samples. Also, a significant variation in bacterial community structures was observed between the rhizosphere regions of C3 and C4 plants grown in PAHs contaminated soils after the 60th and 120th days. Interestingly, 21 different bacterial phyla were observed in the rhizosphere soil samples of C3 and C4 plants, and the dominant bacterial phyla were in the following order: Proteobacteria > Bacteriodetes > Actinobacteria > Verrucomicrobia (Fig. 2). On the other hand, in the unplanted control soils, only four bacterial phyla were recorded in the order of Actinobacteria > Bacteriodetes > Proteobacteria > Verrucomicrobia. To assess the effect of the rhizosphere bacterial community on PAHs removal, a correlation analysis was performed between the bacterial phylum abundance and the PAHs removal percentage for C3 and C4 plants. The analysis revealed a strong positive correlation (*P* ≤ 0.01) between the PAHs removal percentage and the abundance of bacterial phylum Proteobacteria (*r* = 0.91)*,* Firmicutes (*r* = 0.91), Actinobacteria (*r* = 0.87)*,* and Chlorobi (*r* = 0.82)*.* Whereas, the relationship between phylum abundance and PAHs removal was insignificant for Verrucomicrobia and Bacteroidetes (Table S1). At the end of 60 and 120 days, the relative frequency (%) of total bacterial abundance (order level) of both the C3 and C4 rhizosphere samples are given in the supplementary figures (Figs. S1–S6). In both the C3 and C4 rhizosphere samples, the PAHs degrading bacterial genera such as *Arthrobacter*, *Bacillus, Flavobacterium*, *Nocardia*, *Pseudomonas*, *Sphingomonas*, *Stenotrophomonas,* and *Streptomyces* were observed after the 60th day (Fig. 3a,b). Among the bacterial genera, *Stenotrophomonas* recorded the highest relative abundance (%) both in C3 and C4 (27.3 and 27.4%, respectively) rhizosphere soils. Additionally, in the rhizosphere of the C4 plants, other PAHs degrading bacterial genera such as *Agrobacterium*, *Arthrobacter, Bacillus*, *Mycobacterium*, and *Nocardia* were observed but not in the C3 plants rhizosphere samples. However, in the 120th day rhizosphere soil samples, the PAHs degrading bacterial genus *Arthrobacter* dominated with a high relative frequency in both C3 (78.6%) and C4 (68.3%) plants (Fig. 4a,b). Surprisingly, the bacterial genus *Stenotrophomonas* that recorded a high relative frequency percentage in the 60th day rhizosphere soil of both C3 and C4 plants was not recorded in the 120th day rhizosphere sample of C3 plants and occurred only with a low relative frequency percentage in the C4 plants rhizosphere (0.19%). The variation in the PAHs degrading bacterial genera over the rhizoremediation period in the 60th and 120th day rhizosphere samples of plants from C3 and C4 photosystems are given in Figs. S7–S11. A diverse array of PAHs degrading bacterial genera were observed in the 120th day samples but mainly in the C4 plants rhizosphere samples. Also, the bacterial genus *Pseudomonas* was recorded high in the 120th day of C4 plants rhizosphere soil. Even though the emergence of new bacterial genera such as *Acidovorax*, *Aeromonas*, *Brevibacillus*, *Burkholderia*, *Gordonia*, *Microbacterium*, *Micrococcus*, *Nitrosomonas*, *Rhizobium*, *Rhodobacter*, and *Rhodococcus* were observed during the advancement in the experimental duration (120th day) in both C3 and C4 plants rhizosphere, a considerable variation in PAHs degrading bacterial genera was observed between the C3 and C4 plant rhizosphere samples. A strong positive correlation (*P* ≤ 0.05) was observed between the PAHs removal percentage after the 120th day and the relative abundance of the bacterial genera *Arthrobacter*, *Bacillus*, *Nocardia*, *Rhizobium*, and *Xanthomonas* in both C3 and C4 plants rhizosphere. In addition to this, C4 plants also exhibited a significant positive correlation with the bacterial genera *Mycobacterium*, *Pseudomonas Rhodococcus*, and *Sphingomonas* (Table S2).

### Microbial attributes in C3 and C4 rhizosphere samples

**Figure 5:**
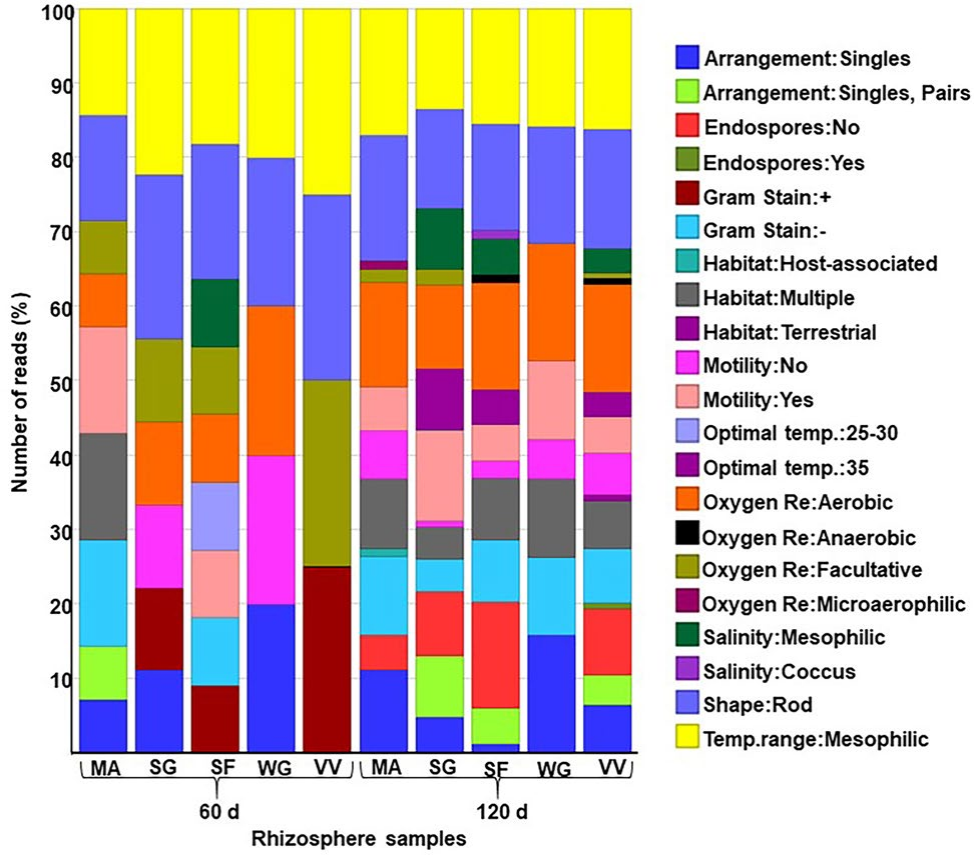
Microbial attributes of 60th and 120th-day rhizosphere soil. The Y-axis represents the number of reads in terms of percentage of different bacterial classes. The X-axis represents rhizosphere samples of MA-maize, SG-Sudan grass, SF-sunflower, VV-vetiver, and WG-wallaby grass at 60th and 120th day.

The summary of microbial attributes describing the cellular features (shape, Gram stain, cell arrangements, endospore formation, and motility), temperature, and their environment (oxygen requirement and salinity) are given in Fig. 5. With regards to Gram staining, most of the rhizosphere samples were observed to be dominated by Gram-negative bacterial strains in 120th day rhizosphere samples except for the 60th day rhizosphere sample of Sudan grass, vetiver, and wallaby grass. Also, the highest percentage of the endospore formers were recorded only in 60th day rhizosphere sample of vetiver. All the rhizosphere samples contained rod-type bacterial cells at both the experimental durations.

Most of the rhizosphere microbes were found to exhibit high motility. Among the rhizosphere samples, organisms with the most significant percentage of motility were recorded for maize and Sudan grass. However, the association of microbial community with the host system was evident only in the 120th day rhizosphere sample of maize (Fig. 5). A wide variation was observed among the different rhizosphere microbial communities regarding their oxygen requirement. Indeed, it was evident from Fig. 5, that the microbial samples from vetiver and sunflower rhizosphere had more anaerobic groups, whereas the others showed facultative and aerobic types.

### Bacterial diversity indices and species richness comparison between C3 and C4 rhizosphere samples

The bacterial species richness and diversity indices were compared between the rhizosphere samples at 60th and 120th day (Table 1). In order to execute the analysis, the sequences detected in the different plant treatments were analyzed based on the bacterial species diversity. The 120th day rhizosphere samples in both the C3 and C4 plants were observed to be more diverse than the 60th day and unplanted samples. The total bacterial species diversity with the highest operational taxonomic units (OTU) was detected in the maize rhizosphere sample of the 60th day (Figure S12). Whereas a reduced number of OTUs were observed in the unplanted soil. The alpha proteobacterial diversity in the 60th-day samples was in the order: maize > sunflower > Sudan grass > wallaby grass > vetiver. At the 120th day, the highest number of OTUs were observed in the vetiver and maize rhizosphere samples, and the diversity was in the order of vetiver > maize > Sudan grass > sunflower > wallaby grass (Figure S13).

**Table 1:** Bacterial diversity indices and species richness comparison between 60 and 120th-day rhizosphere samples.

Bacterial diversity indices	UP	MA 60	SG 60	SF 60	VV 60	WG 60	MA 120	SG 120	SF 120	VV 120	WG 120
Taxa (S)	6	84	39	52	16	22	253	210	209	255	203
Dominance (D)	0.27	0.13	0.16	0.12	0.24	0.28	0.19	0.12	0.23	0.16	0.22
Simpson (1-D)	0.73	0.87	0.84	0.88	0.76	0.72	0.81	0.88	0.77	0.84	0.78
Shannon (H)	1.54	3.22	2.78	3.08	2.00	2.12	3.34	3.64	3.03	3.37	3.09
Evenness (e^H/S)	0.78	0.30	0.41	0.42	0.46	0.38	0.11	0.18	0.10	0.11	0.11
Menhinick	1.81	4.45	3.96	4.44	2.39	2.94	4.72	4.97	4.40	3.88	4.20
Margalef	2.09	14.13	8.31	10.37	3.94	5.22	31.64	27.82	27.03	30.34	26.04
Equitability	0.86	0.73	0.76	0.78	0.72	0.69	0.60	0.68	0.57	0.61	0.58
Fisher alpha	5.40	34.69	24.21	30.56	8.87	13.35	66.83	61.67	56.38	59.27	53.37
Berger-Parker	0.45	0.34	0.37	0.33	0.44	0.52	0.43	0.33	0.47	0.39	0.46
Chao-1	9.00	142.20	82.88	114.3	49	46	334.10	286.10	358.30	320.00	278.10

*MA* maize, *SG* Sudan grass, *SF* sunflower, *VV* vetiver and *WG* wallaby grass, 60–60th-day rhizosphere sample, 120–120th-day rhizosphere sample.

The nonparametric species richness estimator Chao 1 index was used to estimate the number of rare and abundant classes on the 16S rRNA rhizosphere samples. The results indicated that the 60th-day maize sample showed a higher species richness than the other rhizosphere samples. However, on the 120th day, the Chao 1 index was observed to be higher in the sunflower rhizosphere sample, which had less OTU when compared to vetiver, maize, and Sudan grass. Moreover, the bacterial species abundance and evenness, which is reflected in the Shannon and Simpson diversity indices, were observed to be higher in the 120th day in C4 rhizosphere samples.

### Principal component analysis of rhizospheric bacterial diversity in C3 and C4 plants

**Figure 6:**
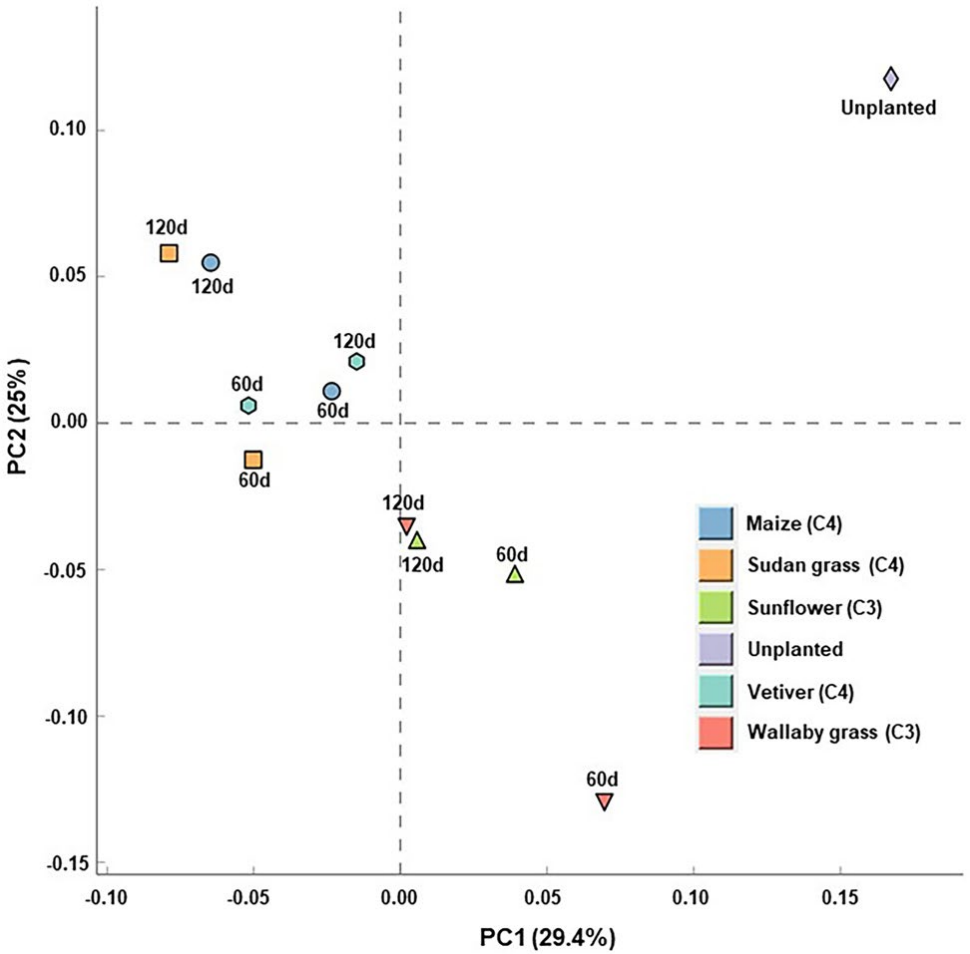
Principal component analysis (PCA) of microbial communities obtained from planted (rhizosphere) and unplanted soils. The data used for the analysis has been normalized to values between 0 and 1.

Principal Component Analysis (PCA) was undertaken with both 60th and 120th-day samples (Fig. 6) to assess the effect of photosynthetic pathways and PAHs degradation ability on the microbial community dynamics. When the photosynthetic pathway was examined as an explanatory variable, the C4 photosynthetic plants such as maize, Sudan grass, and vetiver revealed the highest degree of variation in microbial community dynamics. On the other hand, those plants with the C3 photosynthetic pathway, such as cowpea and wallaby grass, supported relatively less variation in population dynamics except for sunflower in the 60th-day sample. However, on the 120th day, there was a clear difference observed between C3 and C4 plants.

## Discussion

A more significant variation in the rhizodegradation of PAHs was observed between C3 and C4 rhizosphere soils. The per cent rhizodegradation of PAHs by C4 plants was higher than for the C3 plants, which could be due to differences in the root exudates composition of C3 and C4 plants. The concentrations of amino acids and organic acids are reported to be higher in the root exudates of C4 plants, whereas, in the root exudates of C3 plants, the level of carbohydrates are higher^36,37^. Also, C3 and C4 plants differ with regards to the amount and the composition of root exudates^17,19^. The specificity of plant species in facilitating the corresponding microbial populations in the rhizosphere region was documented in earlier research^22^. Similarly, our study shows that the rhizosphere microbial community exhibited a wide variation between the C3 and the C4 plant species. The PAH degradation ability for soil planted with C4 plants was also enhanced when compared to C3 plants, which could be due to greater stimulation of microbial activity by the root exudates of C4 plants resulting in enhanced phytoremediation of soil.

In the present study, the distribution of the major phyla of bacterial community detected using the pyrosequencing technique from the rhizosphere samples includes Proteobacteria, Actinobacteria, Bacteroids, and Verrucomicrobia. These bacterial phyla were reported in the earlier studies as the dominating bacterial phyla in the rhizosphere^38–40^. Members of bacteria belonging to Proteobacteria and Actinobacteria were reported to involve with the degradation of aromatic contaminants^41^. Pyrosequencing analysis makes it possible to analyze even the less abundant and more unknown members of the bacterial community in the rhizosphere region of C3 and C4 plants. Moreover, some functionally important but less abundant bacterial phyla such as Chlorobi, Verrucomicrobia, and Tenericutes were found mainly in 120th day rhizosphere samples. Several studies reported on the involvement of the bacterial species from the phyla Proteobacteria, Firmicutes, and Actinobacteria in PAHs degradation^42–44^. In the present study, a high positive correlation was observed between the PAHs removal percentage and abundance of phyla Proteobacteria, Firmicutes, Actinobacteria, and Chlorobi in the rhizosphere soil. However, the bacterial abundance and the PAHs removal in the unplanted soil was significantly less compared to the rhizosphere of both the C3 and C4 plants in both the stages (60th and 120th day). This confirms that the degradation of PAHs from these soils was mainly due to plant root exudate stimulation of the microbial community in the rhizosphere soil.

Several studies have reported on the greater abundance of the bacterial community and of its composition and structure in rhizosphere soil than in bulk soil^14,22,45^. In this study, more considerable differences were documented between the bacterial populations in the rhizosphere soil than in unplanted soils. Indeed, the proliferation of the diverse bacterial community in C3 and C4 samples was found to be higher at the 120th day when compared to the 60th day rhizosphere sample. The result from the present study demonstrated the role of root exudates in augmenting microbial diversity in rhizosphere soil during different stages of plant development. This outcome may be due to the difference in root exudation patterns associated with root development in the early stages of development. Similarly, previous reports also supported the dynamic variation of the bacterial community composition and structure with the different plant growth stages^46,47^.

Interestingly, the bacterial genus *Stenotrophomonas* that appeared to be dominant in all 60th-day rhizosphere samples exhibited a reduction in the 120th day sample of C4 plants. In contrast, in C3 plants, their sequences were not observed. This result revealed that *Stenotrophomonas* might be sensitive to the metabolites or the by-products formed during PAHs degradation, and therefore, their growth might have inhibited in 120th day rhizosphere samples. A similar result was observed with *Stentrophomonas maltophilia* VUN 10003, which exhibited inhibition of growth and degradation of PAHs, benzo[*a*]pyrene, and dibenz[*a,h*]anthracene due to the accumulation of metabolites in the culture medium^48^. On the other hand, the bacterial genus *Pseudomonas* was reported to have a high PAHs degradation ability without any reduction in its growth during the degradation process^49^. The strain *Pseudomonas aeruginosa* NY 3 was reported to produce several rhamnolipids—a biosurfactant that improves the degradation of high molecular weight PAHs with the synergistic improvement in its growth on PAHs contamination^50^. Similarly, in the present study, a higher percentage of *Pseudomonas* sequences were observed in all 120th day rhizosphere samples. This result suggests that the genus *Pseudomonas* can better withstand PAHs and their metabolites, which might be due to the production of biosurfactants that aid in the efficient degradation of PAHs without affecting the bacterial growth.

A more significant difference in the rhizosphere microbial attributes was recorded in the present study between C3 and C4 plants and also with the duration of growth in the PAHs contaminated soil. Many researchers previously reported PAHs degrading bacterial species like *Burkholderia cocovenenans*, *Sphingobium barthaii*, *Rhizobium petrolearium*, *Neptunomonas naphthovoran,* and *Pseudomonas* sp. fall under the rod-shaped Gram-negative bacterial type^51–54^. Likewise, in the present study, most of the rhizosphere bacterial species belonged to the rod-shaped and Gram-negative groups of bacteria. The initial step of aerobic PAH metabolism involves ring-hydroxylation by PAHs ring-hydroxylating—dioxygenase enzymes (PAH-RHD) with an *α* subunit. The Gram-negative bacterial genera, such as the *Alcaligenes*, *Burkholderia*, *Commamonas*, *Novosphingobium*, *Polaromonas*, *Pseudomonas, Ralstonia,* and *Sphingobium* reported to possess PAH-RHD mediated PAHs degradation^55^. Motility of bacteria is one of the essential factors in determining the rhizodegradation of contaminants in the soil that are distant from the rhizosphere region^56^. In the present study, most of the rhizosphere bacterial species exhibited a high percentage of motility, especially with the 120th day rhizoremediated soil.

The estimated number of OTUs was less in the unplanted PAHs contaminated soil than in the rhizoremediated samples, which might emphasize that the C3 and C4 plants enhanced the bacterial species richness in the rhizoremediated soil by reducing the toxicity of PAHs and promoting the growth of the bacterial community. The results of the present study was comparable with the earlier research on the reduced species richness and bacterial diversity in the heavy metal contaminated soil^52^. All the C4 plants except the vetiver exhibited a higher level of OTUs than the C3 plants at the 60th day rhizosphere sample. This difference in the level of OTUs in the vetiver rhizosphere is due to the slow growth of the vetiver slips at the early stage of the plant development. Also, the time required by the vetiver slips to get acclimatized to PAHs contaminated soils was higher^20^. However, at 120th-day, a clear difference in OTU and alpha diversity was observed between C3 and C4 plant rhizosphere samples. Earlier studies reported on the conservation, restructuring, and expansion of the rhizosphere microbiome during the plant growth due to the changes in the plant root exudate pattern in *Arabidopsis*, maize, wild oats, and switchgrass^40,57–59^. This suggests that the higher bacterial diversity observed in the 120th day rhizosphere samples could be due to the addition of plant root products to the PAHs contaminated soil, which then promoted the growth of diverse bacterial species, especially in vetiver and maize.

## Conclusion

This study confirms the role of plants in promoting microbial populations, which in turn facilitates PAHs degradation. The microbial diversity between the C3 and C4 plants increased with their growth stage. Among the plant species, the diversity of PAHs degrading bacterial genera was higher in C4 plants compared to the C3 plants. Also, a strong positive correlation was observed between PAHs removal and the abundance of bacterial phyla such as Proteobacteria, Firmicutes, and Actinobacteria. Therefore, this study provides novel insights towards understanding of the possible role of plant photosystems (C3 and C4 plants) in determining the structure and composition of the plant rhizosphere microbiomes that are actively involved in the degradation of PAHs in contaminated soils.

## Materials and methods

### Experimental conditions

The PAHs contaminated soil was collected from a landfill site in Dublin, South Australia (latitude − 34.445, longitude 138.356). The soil was air-dried, sieved (< 2 mm). Soil texture, pH, EC, and dissolved organic carbon (DOC) and water holding capacity were measured using standard procedures^5^, and their corresponding values were given in supplementary tables S3 and S4. For rhizoremediation experiments, plants such as Cowpea (*Vigna unguiculata*), sunflower (*Helianthus annus*), and wallaby grass (*Austrodanthonia caespitosa*) with C3 photosystems and maize (*Zea mays*), Sudan grass (*Sorghum sudanense*), and vetiver (*Vetiveria zizanoides*) with C4 photosystems were chosen. As previously described in our study by Sivaram et al.^5^, the seeds were surface sterilized and sown in PAHs contaminated soil in three replications and maintained in greenhouse conditions with the experimental durations of 60 and 120 days^21^. Unplanted PAHs contaminated soil served as a control treatment.

### PAHs determination

A 20 g of the PAHs contaminated soil sample was used to determine the initial concentration of PAHs. The extraction of PAHs was done as per the procedure described in our previous study^5^. In short, the concentration of PAHs was analysed using the Zorbax Eclipse Colum XDB- C18 fitted to high performance liquid chromatograph (Agilent Technologies 1200). PAHs were detected using UV-fluorescence detector (FLD) with excitation and emission of 297 and 405 nm, respectively. A certified standard mixture of PAHs (Supelco, Bellefonte, PA, USA) was used for external calibration. QA/QC was carried out with a solvent blank, and the known standard was injected after every ten samples. The PAHs concentration in soil extracts was determined and expressed on a soil dry weight basis.

### Soil DNA extraction, PCR, and 16S rRNA sequencing

The rhizosphere soil samples from C3 (cowpea, sunflower, and wallaby grass) and C4 plants (maize, Sudan grass, and vetiver) and unplanted control soil samples were collected at the end of 60 and 120 days. Due to the poor PAHs degradation ability, cowpea plants were excluded from 120 days of experimental studies. The soil genomic DNA was isolated using Mo-Bio Power Soil Kit. The genomic DNA from the three replicates of each plant species were pooled. The rationale for DNA pooling was based on the Linear mixed effects model by R programming on the response of microbial activity measured in terms of dehydrogenase activity (DHA) on the experimental duration and PAHs degradation by C3 and C4 plants. The results of the model were provided in the supplementary table (Table S4). It is evident from the analysis that there was no significant difference in the DHA among the replicates as demonstrated in our previous study^20^ involving the rhizosphere soils of C3 and C4 plants. Therefore, the composite genomic DNA from each treatment was further used to analyze the bacterial communities by 16S rRNA gene pyrosequencing^60–62^. PCR amplification and sequencing were done at the Australian Genome Research Facility (AGRF), Brisbane, Australia. Amplicons were generated for genomic DNA using AmpliTaq Gold 360 Master Mix (Life Technologies, Australia) and the 16S primers 27F (AGAGTTTGATCMTGGCTCAG) and 519R (GWATTACCGCGGCKGCTG). The thermal cycler programming was 94 °C for 3 min; 94 °C for 45 s, 50 °C for 1 min, 72 °C for 1 min (34 cycles) followed by a final extension at 72 °C for 7 min. The 454 A and B adaptors were fused with the amplicons. The concentration and length of the amplicons were measured via fluorometry, followed by normalization and measured with qPCR, and again normalized and pooled in equimolar ratios. The resultant amplicon libraries (equimolar mixture) were used for pyrosequencing in the 454 Genome Sequencer FLX platform using XLR70 chemistry (Roche Applied Biosystems, Australia). Further sequence analysis was carried out with the FASTA file obtained for each sample.

### Computational analysis

The DNA sequences were uploaded in the Meta Genomics Rapid Annotation using Subsystem Technology (MG-RAST) pipeline server version 3.3 (https://metagenomics.anl.gov)^63^. The taxonomic analysis was undertaken using BLASTX and compared the assembled reads against the SEED database tool^64^ of MG-RAST and by BLASTN against the Ribosomal Database Project (RDP)^65^ The reads were processed with a maximum E value of 10^–5^ and a minimum alignment length of 50 bp^66^. The default parameters of the MG-RAST were used for the taxonomic and functional analysis of the sequences. The metagenome ID generated from MG-RAST for the samples submitted are 4552516, 4552517.3, 4,552518.3, 4552519.3, 4,552,520.3, 4552521.3, 4552523.3, 4570273.3, 4570274.3, 4570275, 4570276, and 4570278. The phylum comparison between the experimental duration was carried out using Krona plugin in MG-RAST. There is no variation in the metagenomic profiles of unplanted samples in the 60th and 120th days. Therefore the metagenomic data of unplanted soil samples after 120 days experiment alone was considered for further analysis. The PAHs degrading bacterial genera were selected from MG RAST analysis based on the reports of the earlier studies^19,67–70^.

Statistical Analysis of Metagenomic Profiles (STAMP) package was used to perform principal component analysis (PCA) and profile bar plot to compare the variation in bacterial class with plant duration. The tab-separated files from the MG-RAST were used to create stamp profile files. Firstly, a profile bar plot at selected PAHs degrading bacterial genus level was generated between C3 and C4 plants with 60th and 120th day rhizosphere samples. Metagenomes using two samples test, statistical test: two-sided Fisher’s exact test statistic with the Difference between proportions (DP): Asymptotic—Continuity correction (CC) confidence interval method and Benjamini—Hochberg False discovery rate (FDR) multiple test correction were performed. Also, the deviation in the bacterial genera between 60 and 120th day rhizosphere soil samples were also analyzed by a profile bar plot, adopting the same statistical test. Secondly, a PCA plot was generated using the genus-level metagenome of both the C3, C4 plants, and unplanted samples using STAMP analysis by adapting the procedure described by Parks et al.^71^. The rare fraction curve based on the bacterial diversity was performed with Paleontological statistics (PAST3) software using stamp profile files (spf).

The Biological Observation Matrix (BIOM) data file format generated by the inputs from the Quantitative Insights into Microbial Ecology (QIIME) plugin of MG-RAST was used for analysis with Metagenomic Analyzer (MEGAN version 5.10.3). The hierarchical analysis of bacterial species in both unplanted and planted soils down to the phylum level was performed. The microbial attributes were analyzed for the rhizosphere bacterial samples using MEGAN with a percentage scale^72^.

## Supplementary information

Supplementary file1.
