# Supplementary material for: Rhizodegradation of PAHs differentially altered by C3 and C4 plants
Source: Sci Rep. 2020 Sep 30;10:16109. doi: 10.1038/s41598-020-72844-4 (PMC7527560; doi:10.1038/s41598-020-72844-4)
Supplement: Supplementary file 1 — Supplementary file1. [file 41598_2020_72844_MOESM1_ESM.docx]

**Rhizodegradation of PAHs differentially altered by C3 and C4 plants**

Anithadevi Kenday Sivaram^1,2,3^, Suresh Ramraj Subashchandrabose^1,2,3^, Panneerselvan Logeshwaran ^1,2,3^, Robin Lockington^2,3^, Ravi Naidu^1,2,3^, Mallavarapu Megharaj ^1,2,3^*

^1^Global Centre for Environmental Remediation, The University of Newcastle (UoN), University Drive, Callaghan NSW 2308, Australia.

^2^Cooperative Research Centre for Contamination Assessment and Remediation of Environment, Advanced Technology Centre, The University of Newcastle (UoN), University Drive, Callaghan NSW 2308, Australia.

^3^Centre for Environmental Risk Assessment and Remediation, University of South Australia, Adelaide, SA, Australia.

*Corresponding author – Mallavarapu Megharaj

Email Id: megh.mallavarapu@newcastle.edu.au.


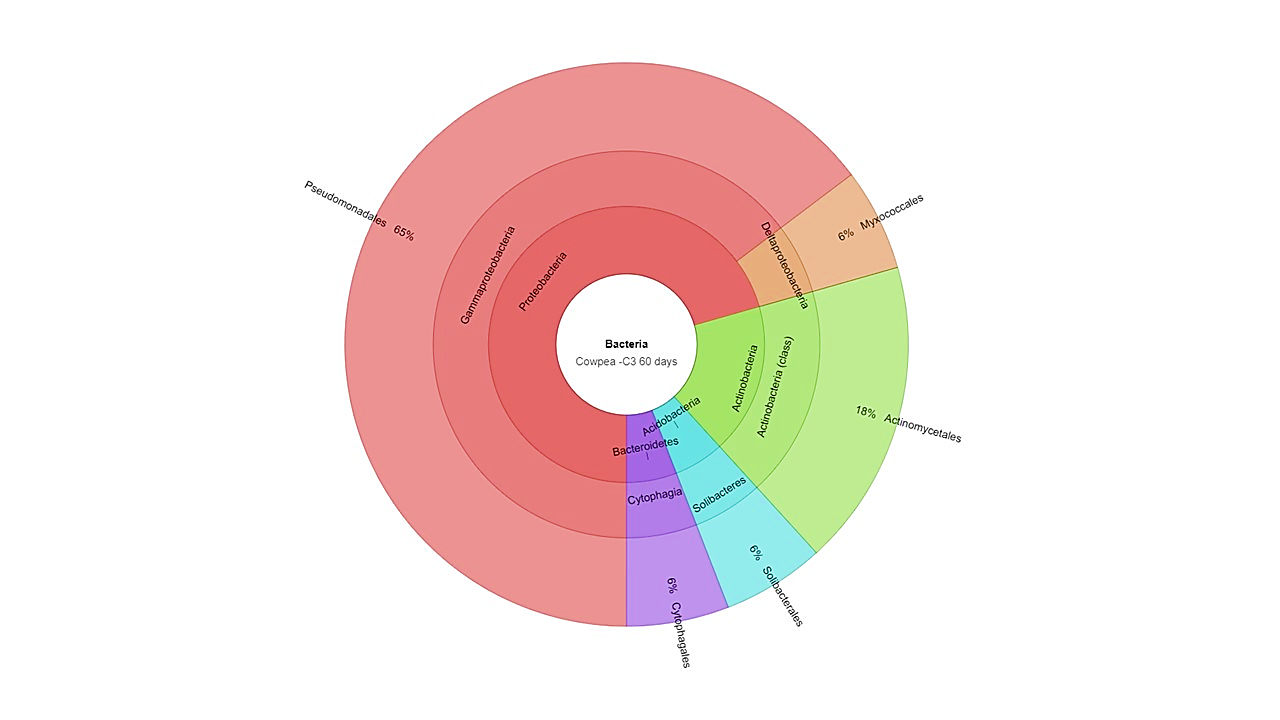


**Figure S1**. Bacterial abundance (order level) in the rhizosphere of cowpea after the 60^th^ day experiment.


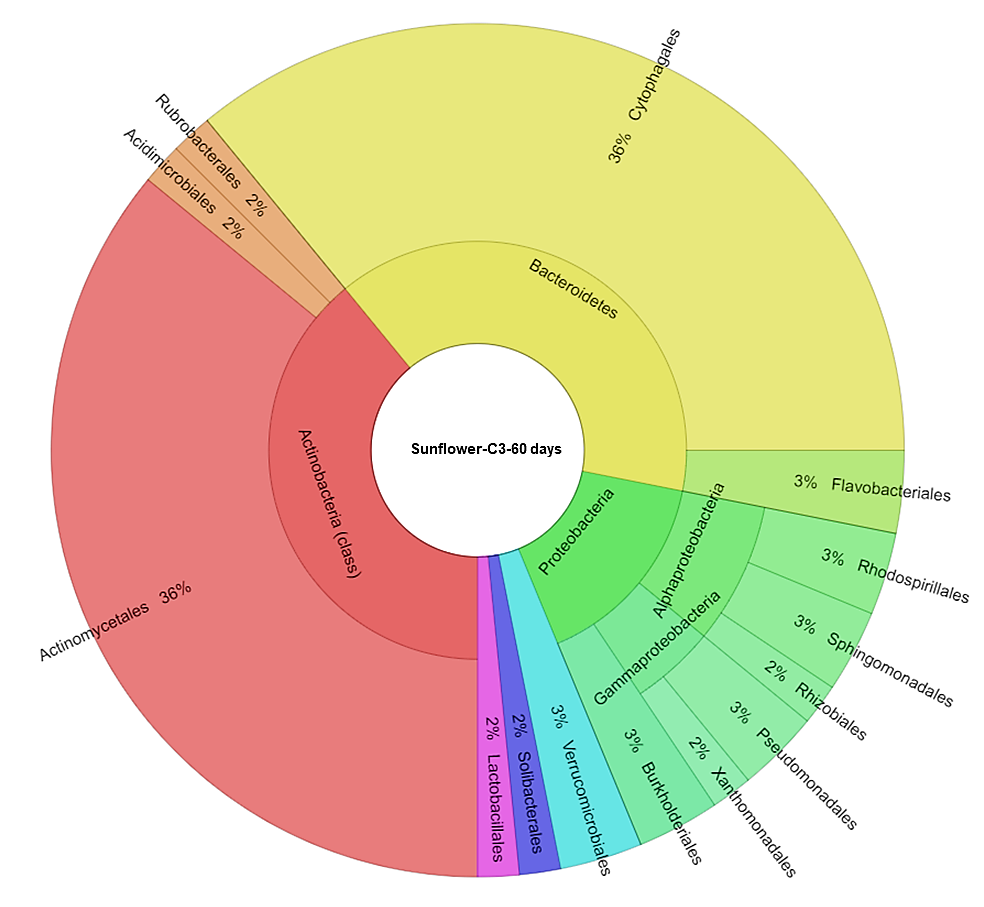


**(2a)**


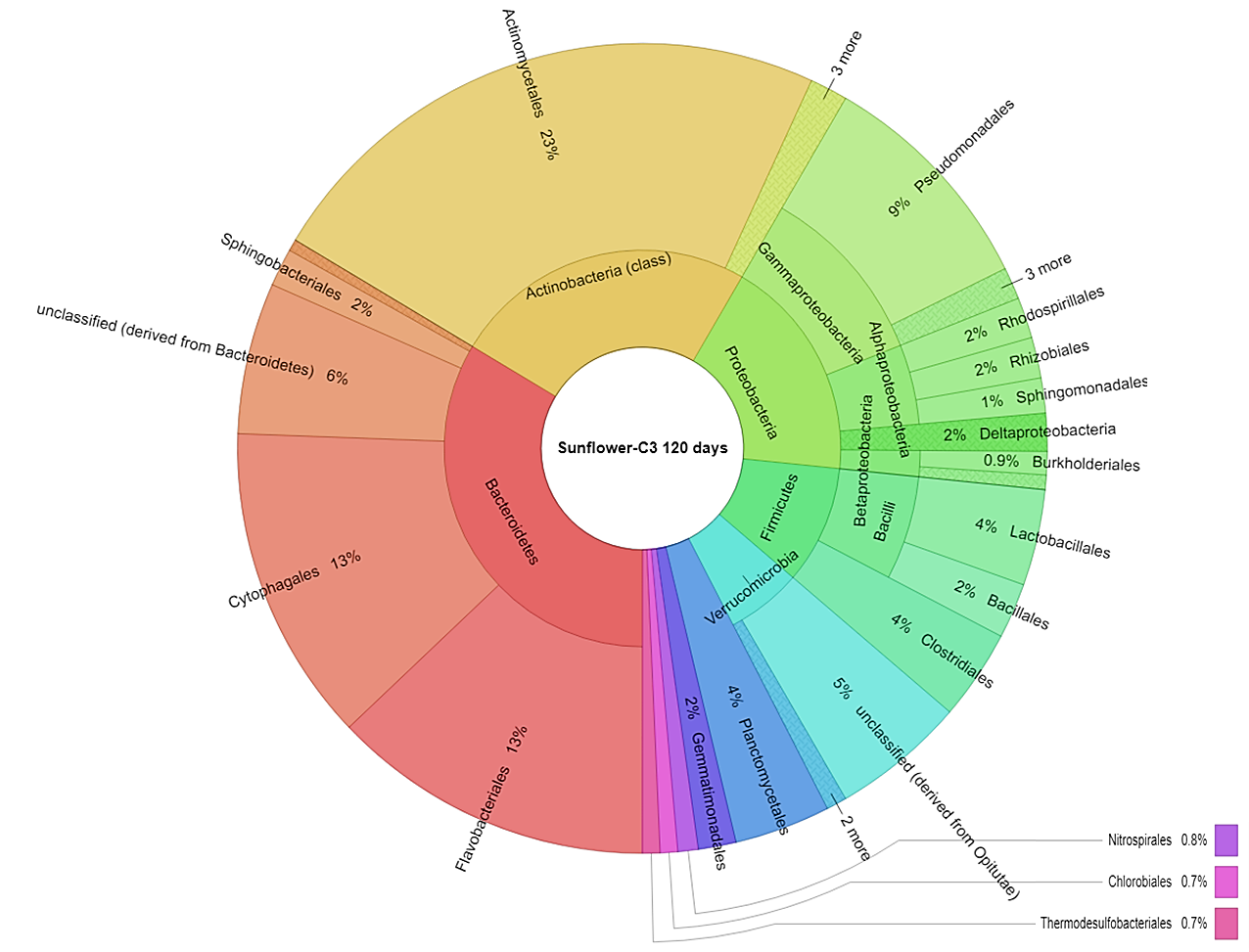


**(2b)**

**Figure S2. A comparison of** bacterial abundance (order level) between (2a) 60, and (2b) 120^th^ day rhizosphere samples of Sunflower.


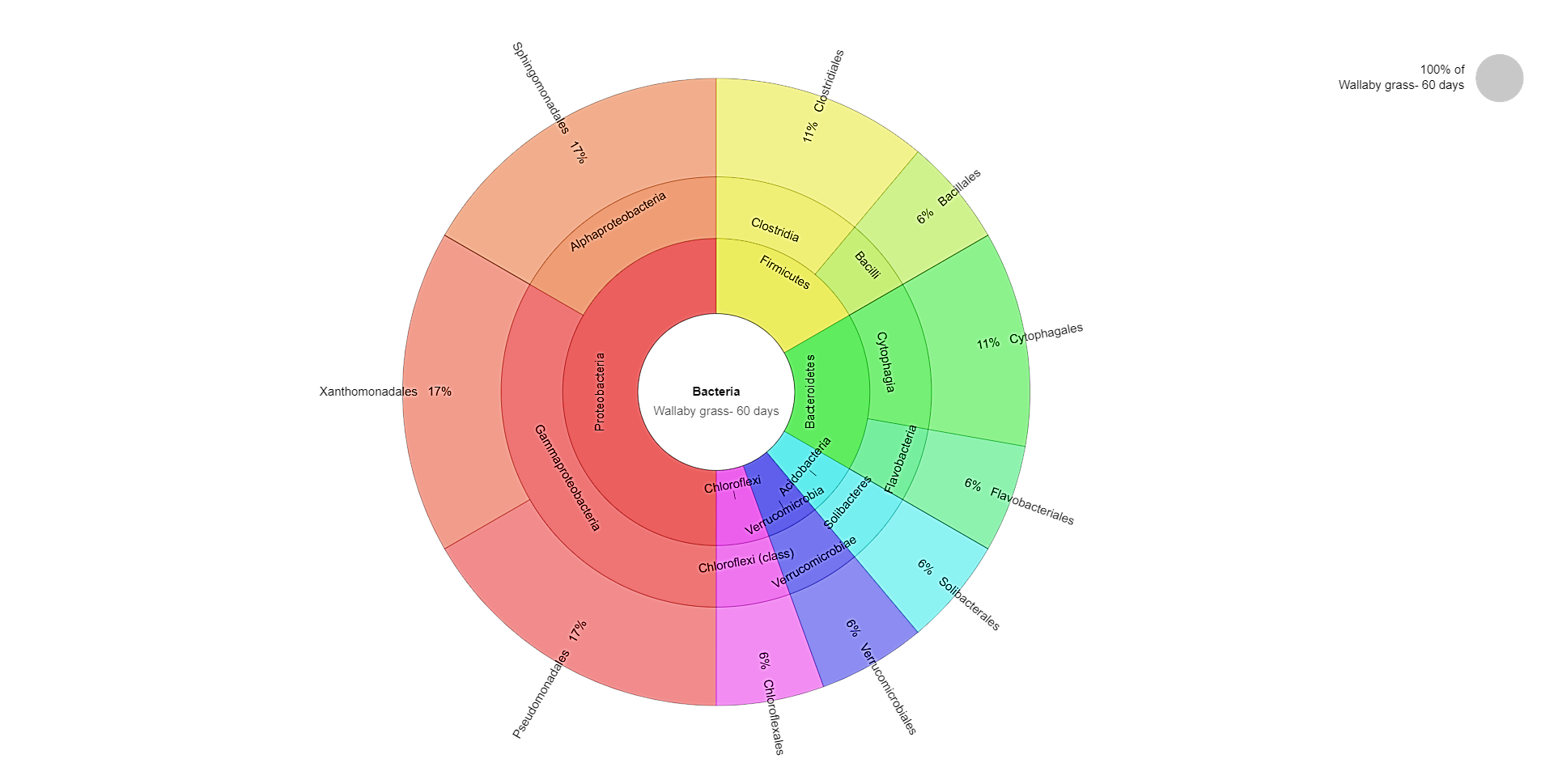


**(3a)**


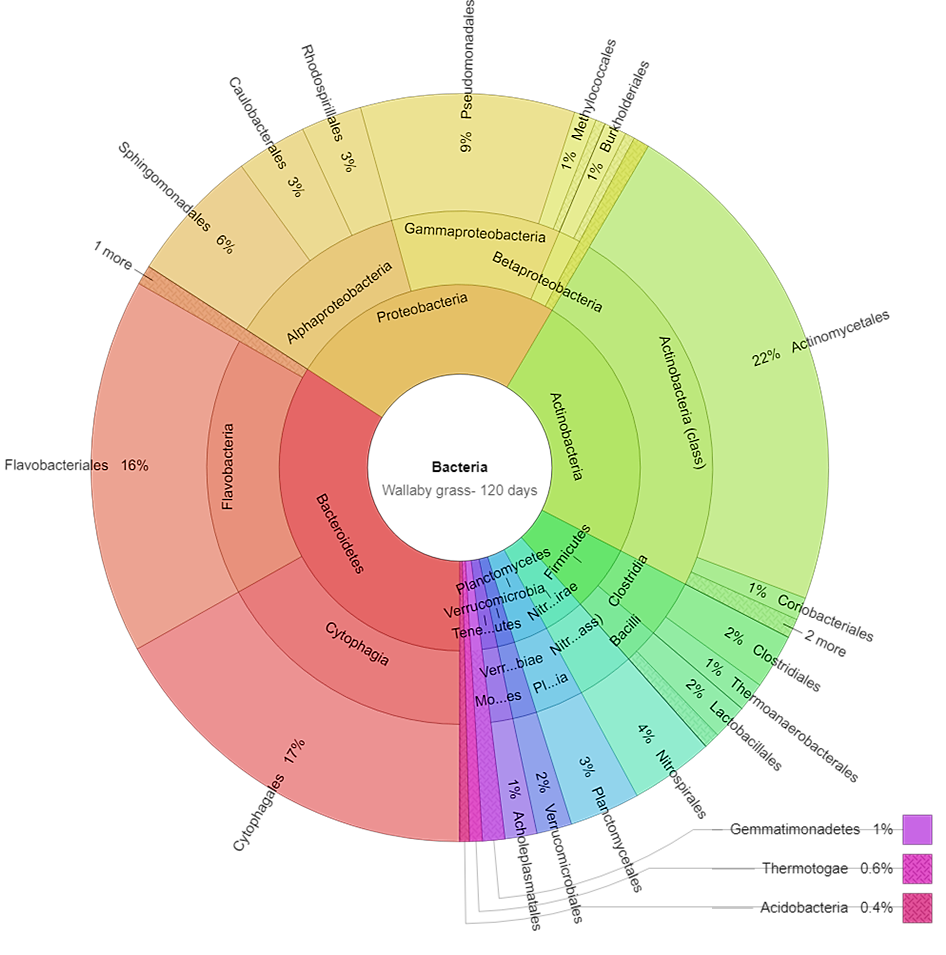


**(3b)**

**Figure S3.** Comparison of bacterial abundance (order level) between (3a) 60, and (3b) 120^th^ day rhizosphere sample of Wallaby grass.

**
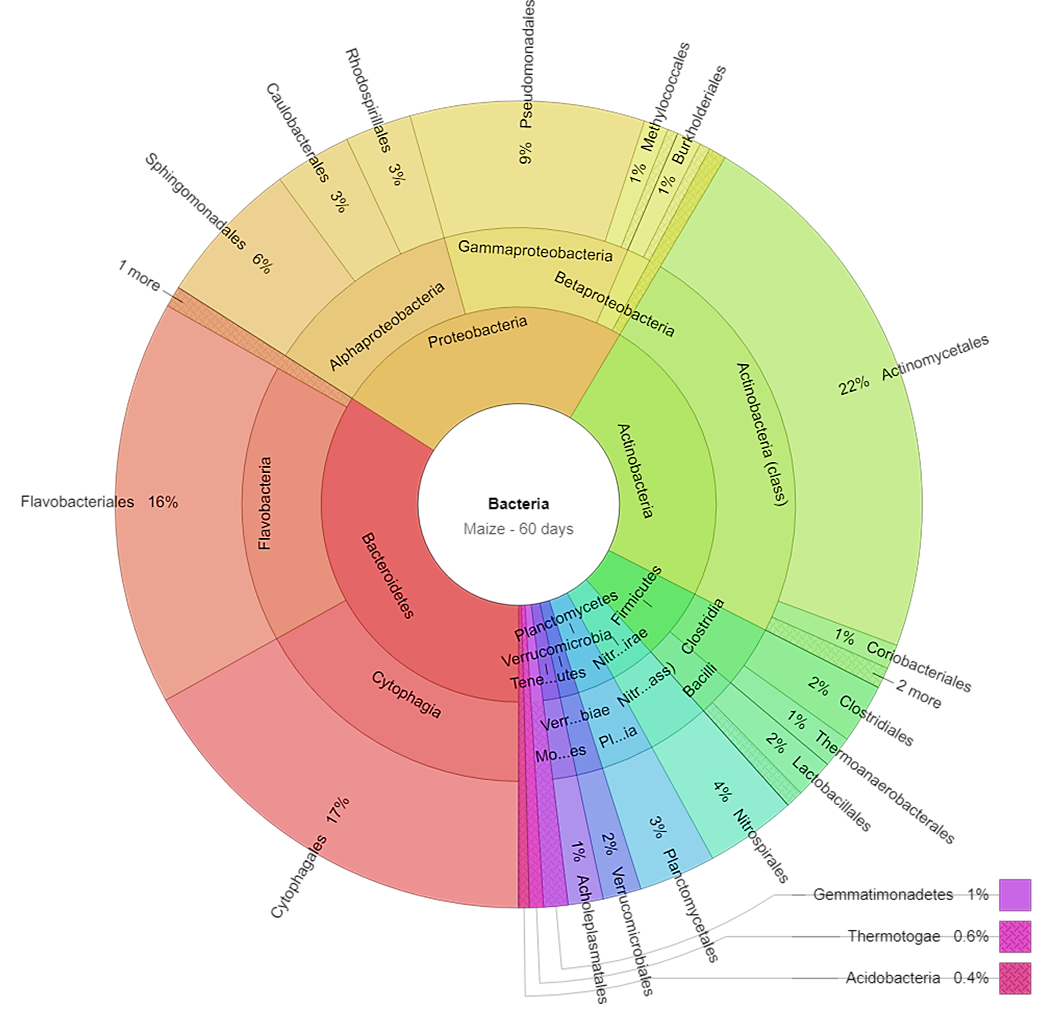
**

**(4a)**

**
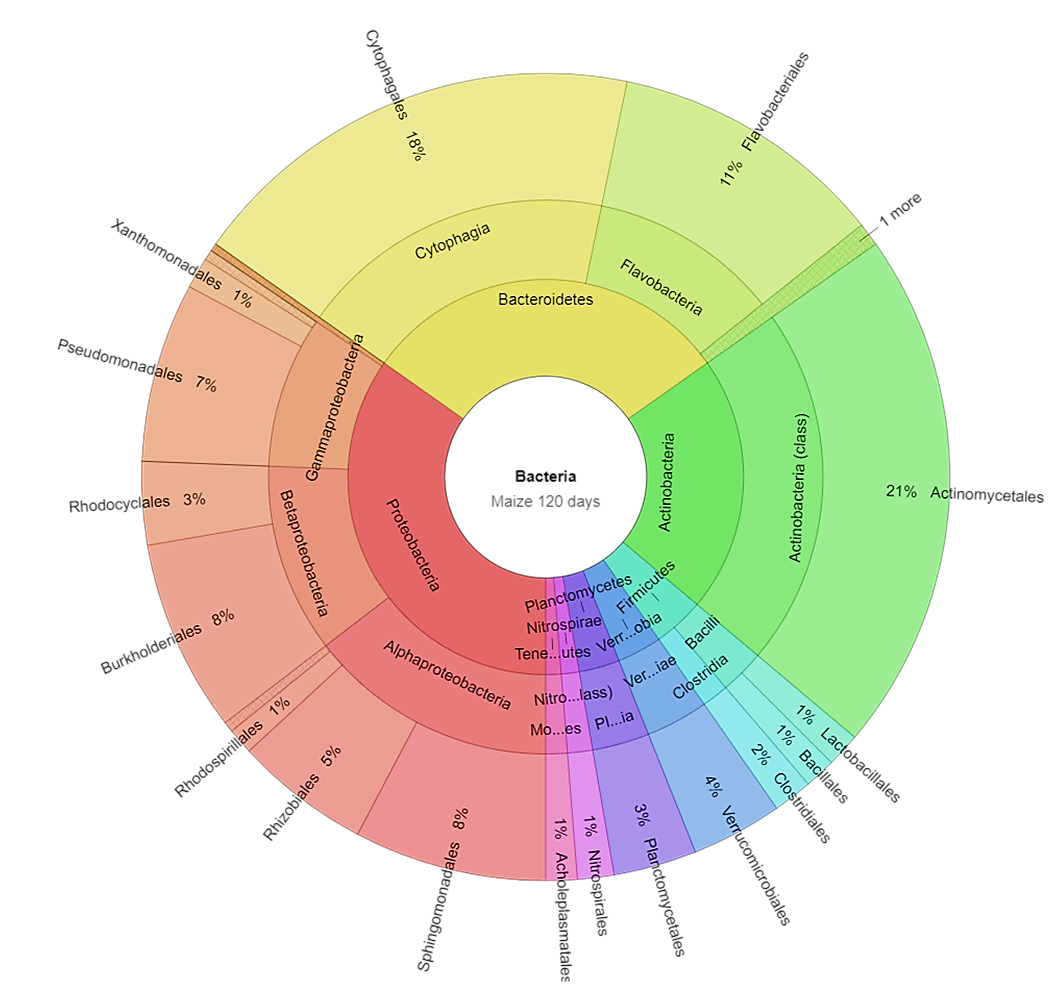
**

**(4b)**

**Figure S4. Comparison of** bacterial abundance (order level) between (4a) 60, and (4b) 120^th^ day rhizosphere sample of Maize.

**
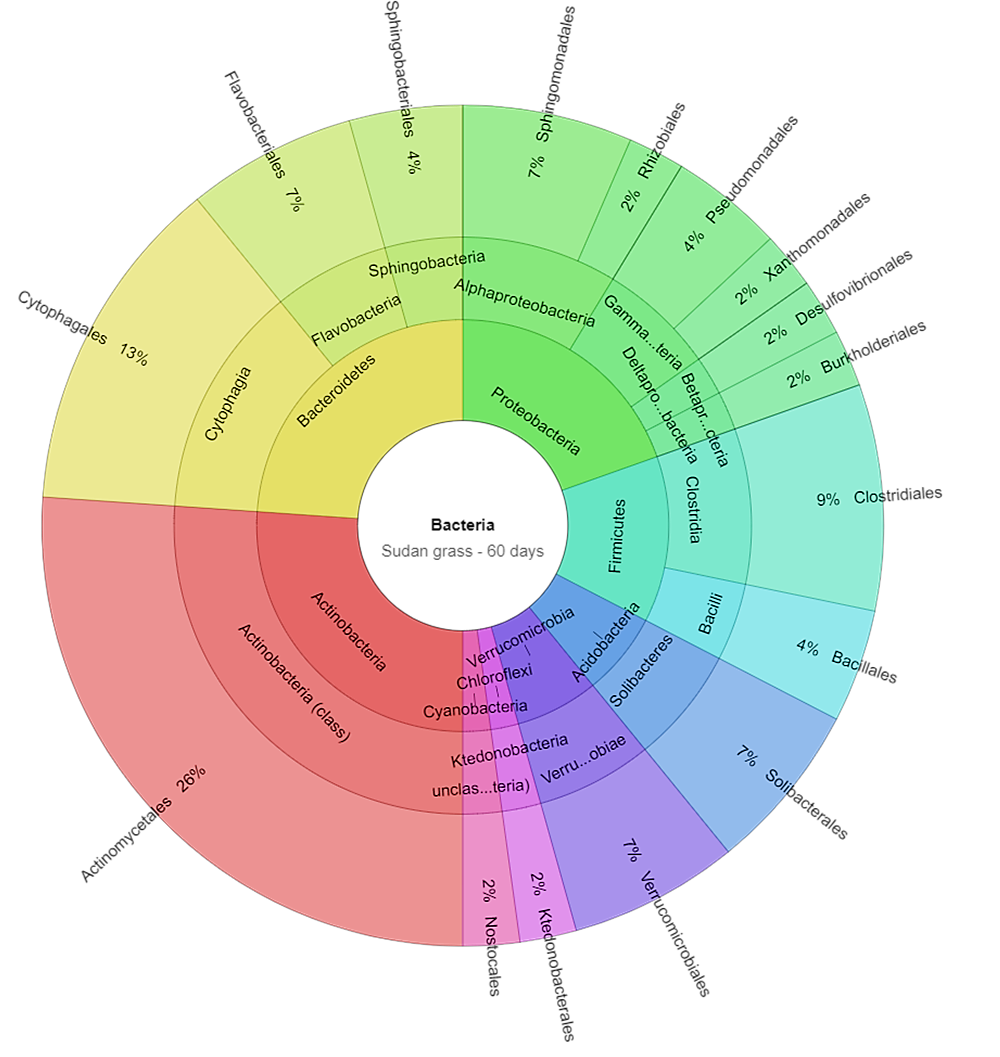
**

**(5a)**

**
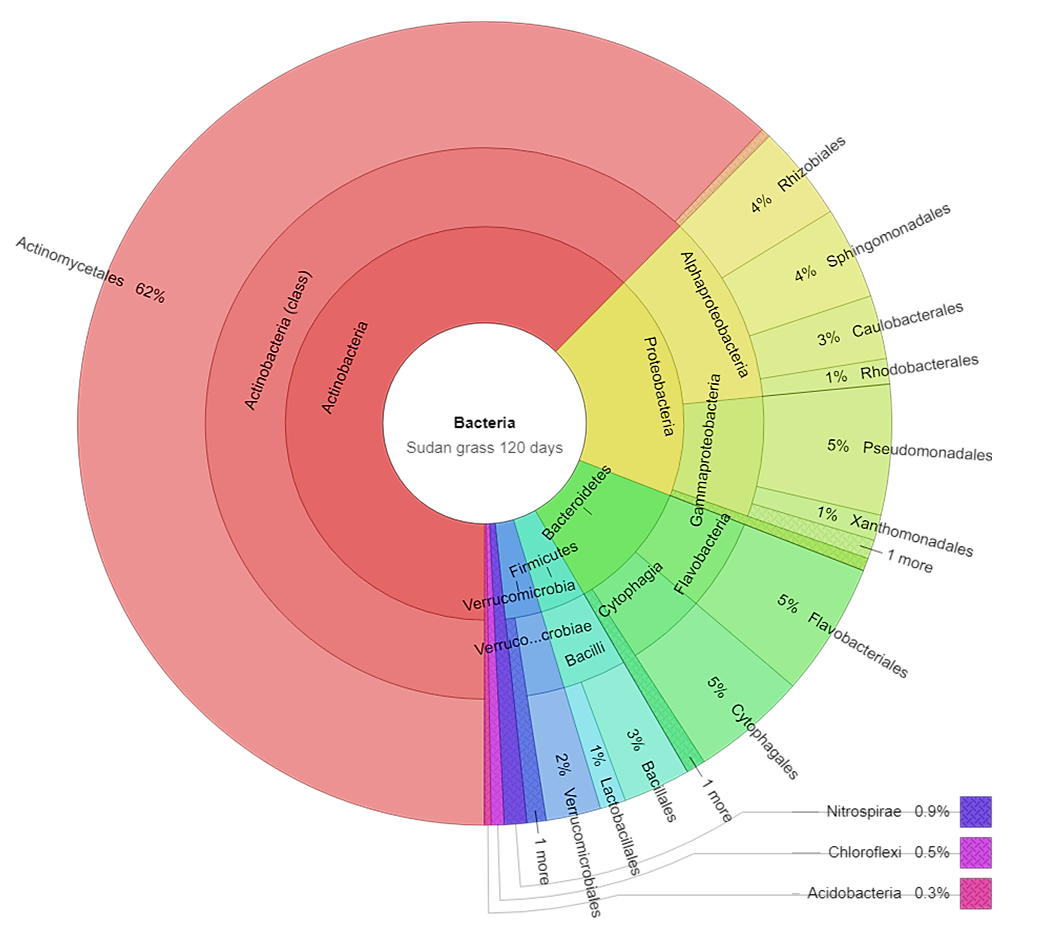
**

**(5b)**

**Figure S5.** Comparison of bacterial abundance (order level) between (5a) 60, and (5b) 120^th^ day rhizosphere sample of Sudan grass.


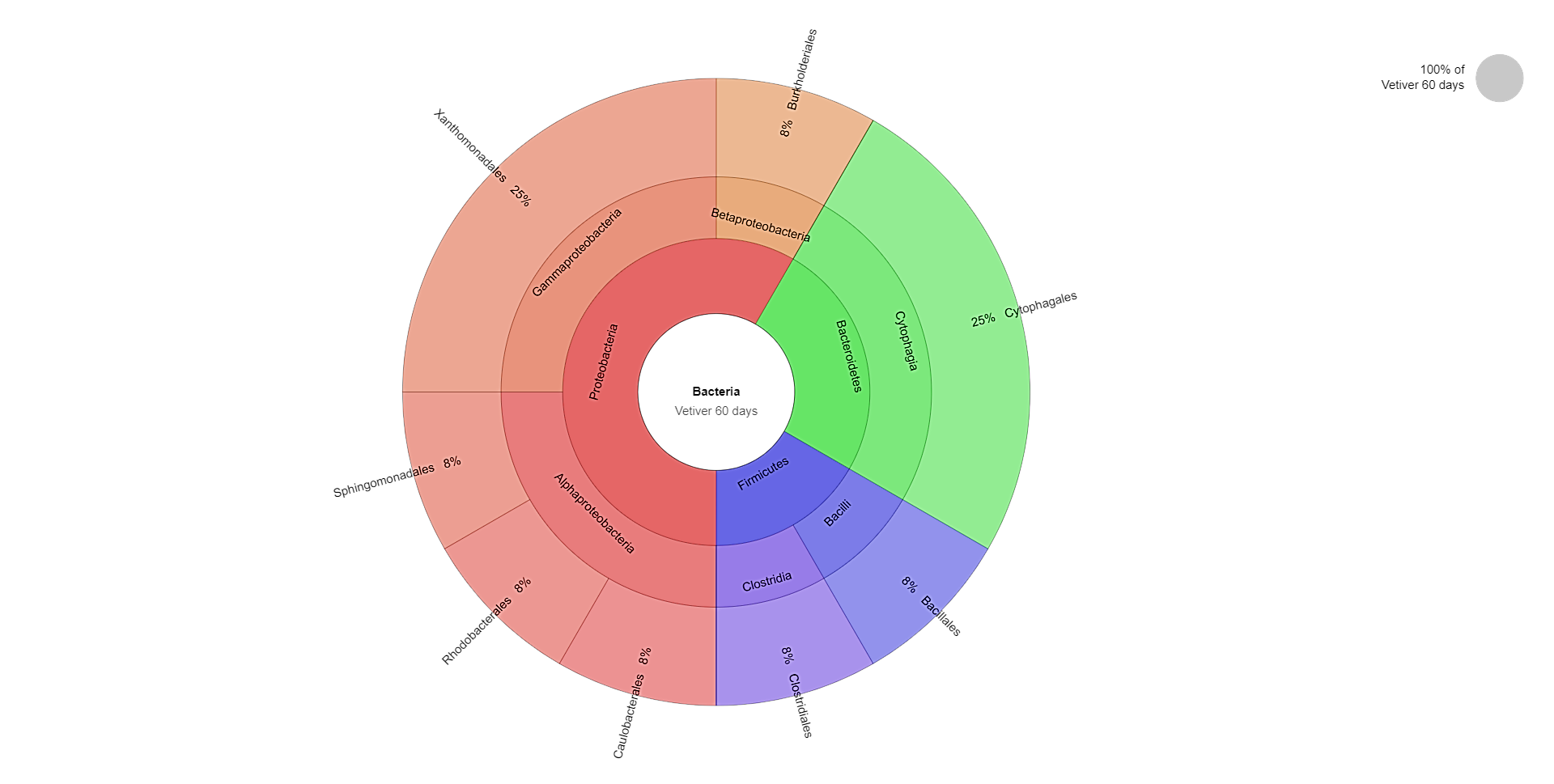


**(6a)**


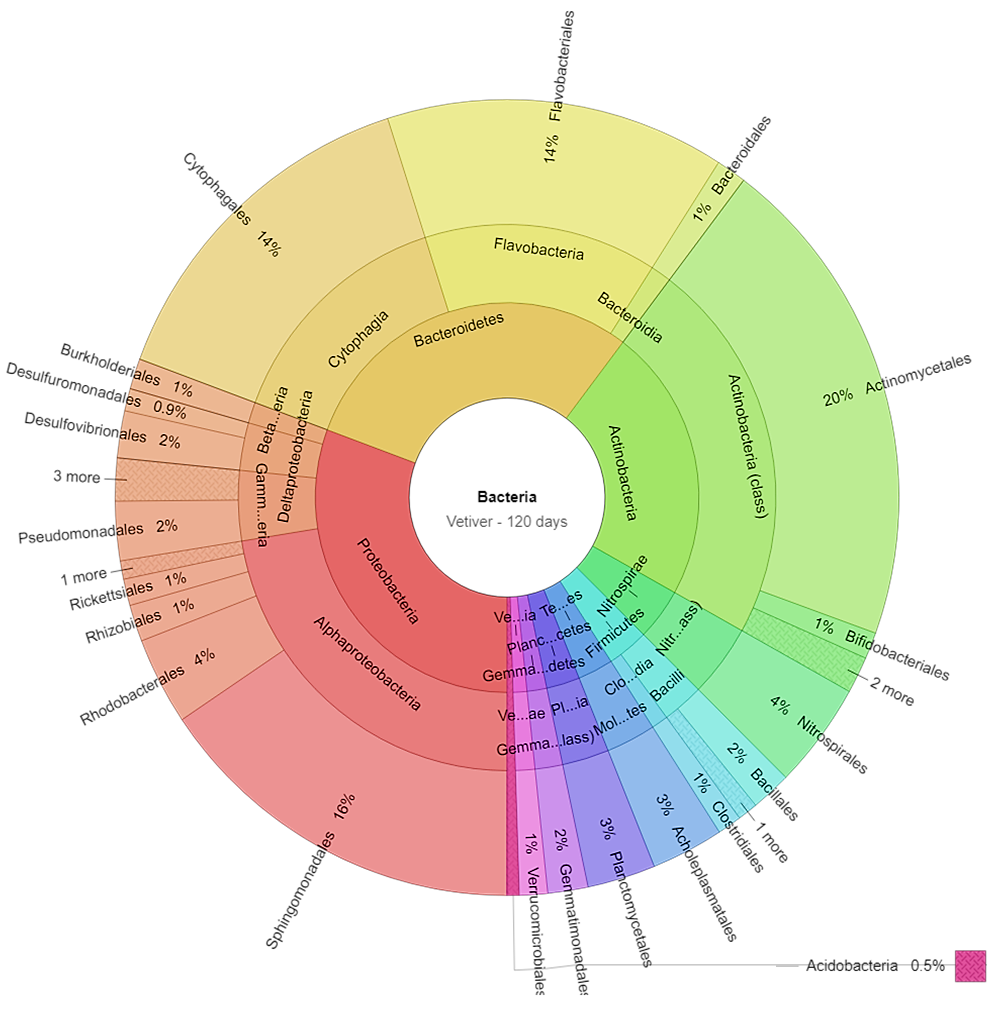


**(6b)**

**Figure S6.** Comparison of bacterial abundance (order level) between (6a) 60 and (6b)120^th^ day rhizosphere sample of Vetiver.


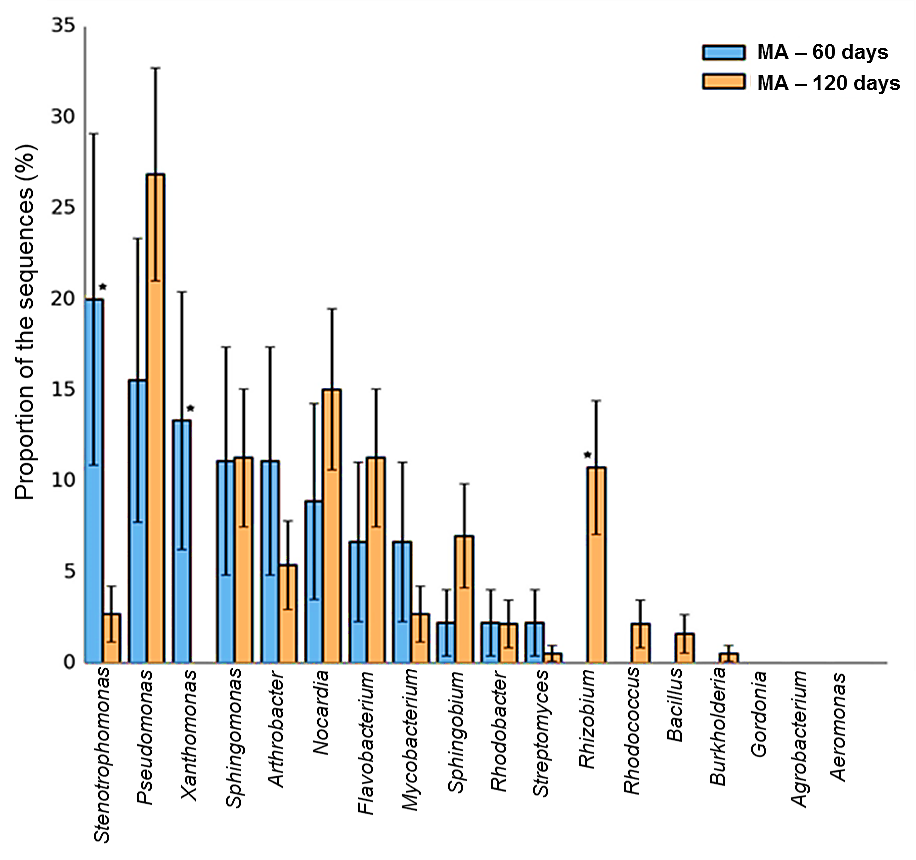


**Figure S7**. Variation in PAHs degrading bacterial genera of maize (MA) in 60^th^ and 120^th^ day rhizosphere soil sample. * - Statistical significance at *p* ≤ 0.05


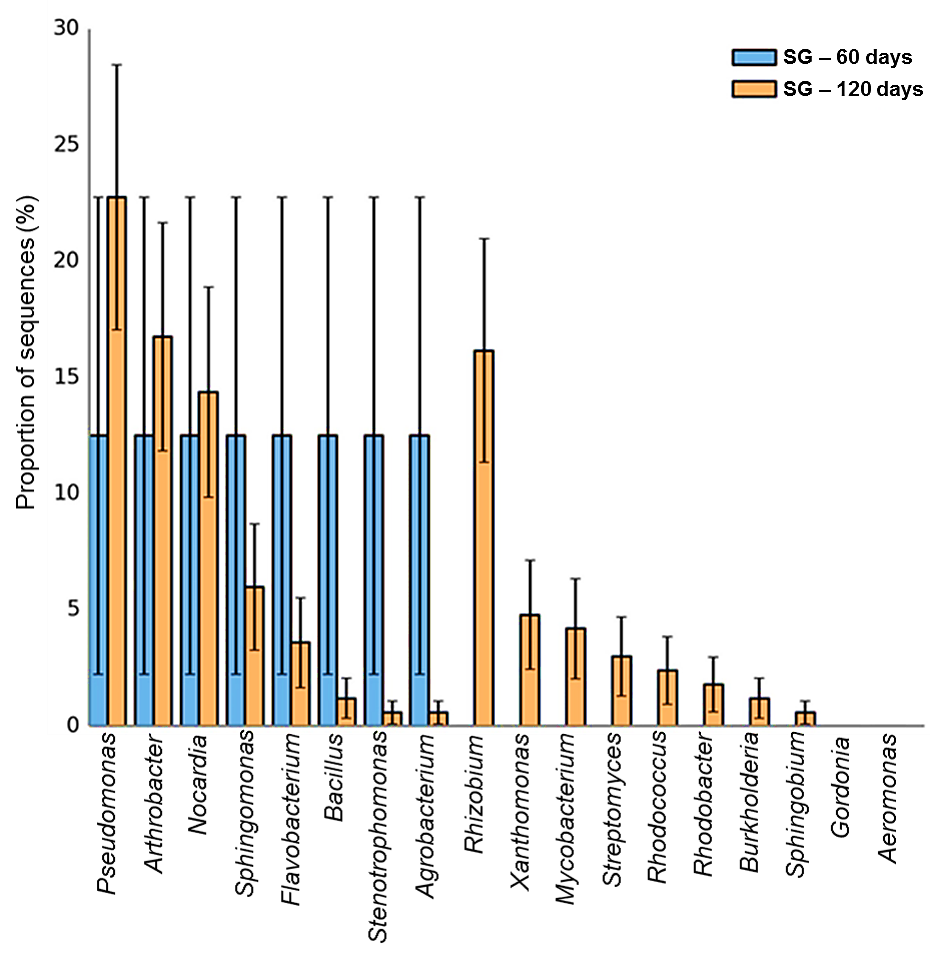


**Figure S8**. Variation in PAHs degrading bacterial genera of Sudan grass (SG) in 60^th^ and 120^th^ day rhizosphere soil sample. * - Statistical significance at *p* ≤ 0.05

**
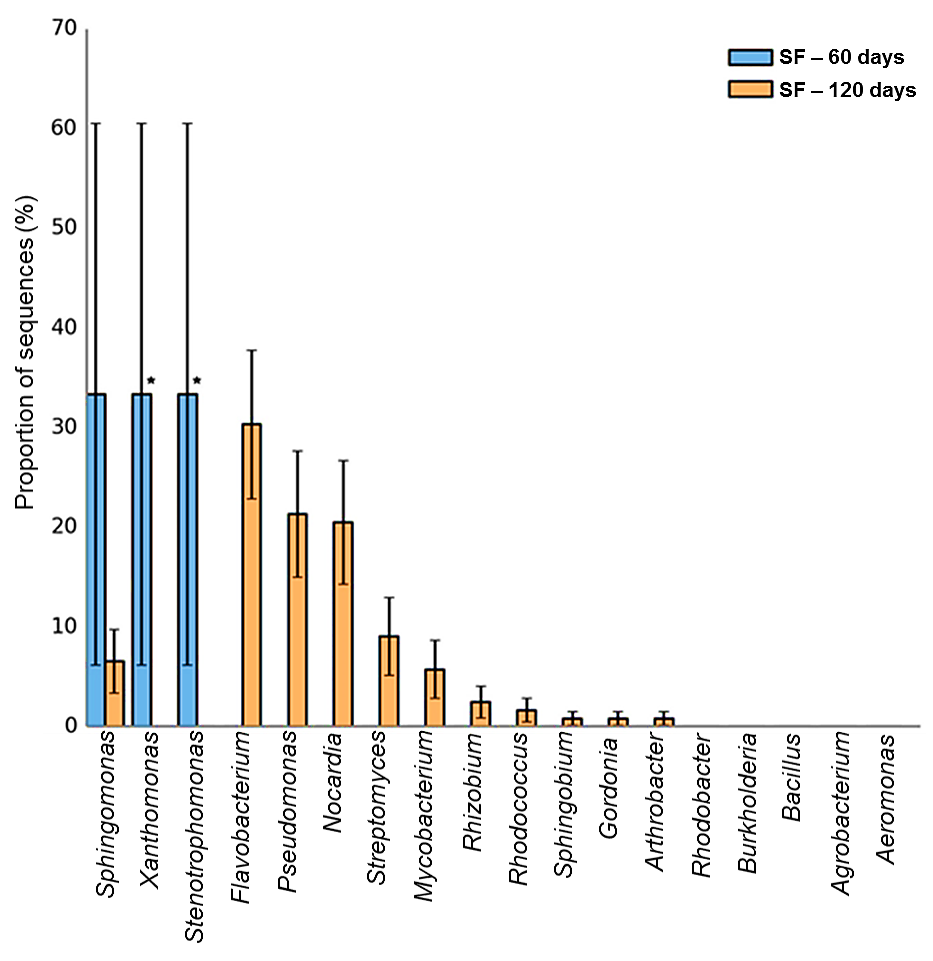
**

**Figure S9**. Variation in PAHs degrading bacterial genera of sunflower (SF) in 60^th^ and 120^th^ day rhizosphere soil sample. * - Statistical significance at *p* ≤ 0.05

#
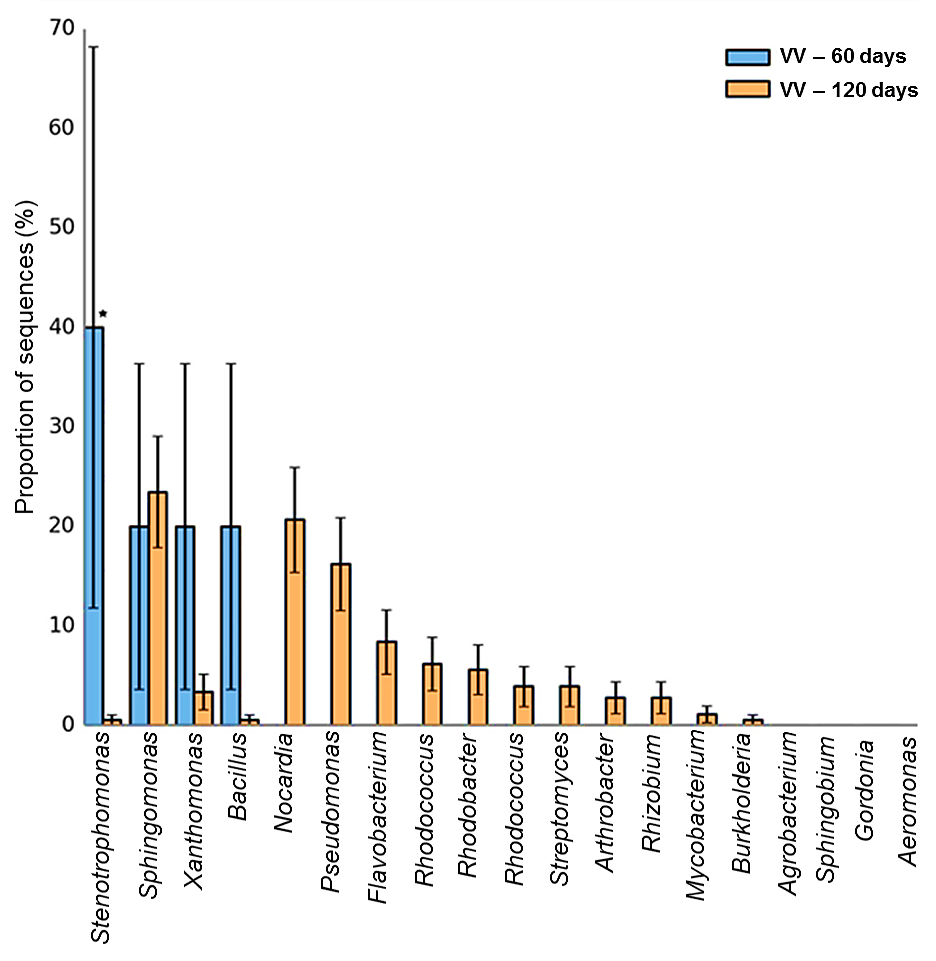


**Figure S10**. Variation in PAHs degrading bacterial genera of vetiver (VV) in 60^th^ and 120^th^ day rhizosphere soil sample. * - Statistical significance at *p* ≤ 0.05

**
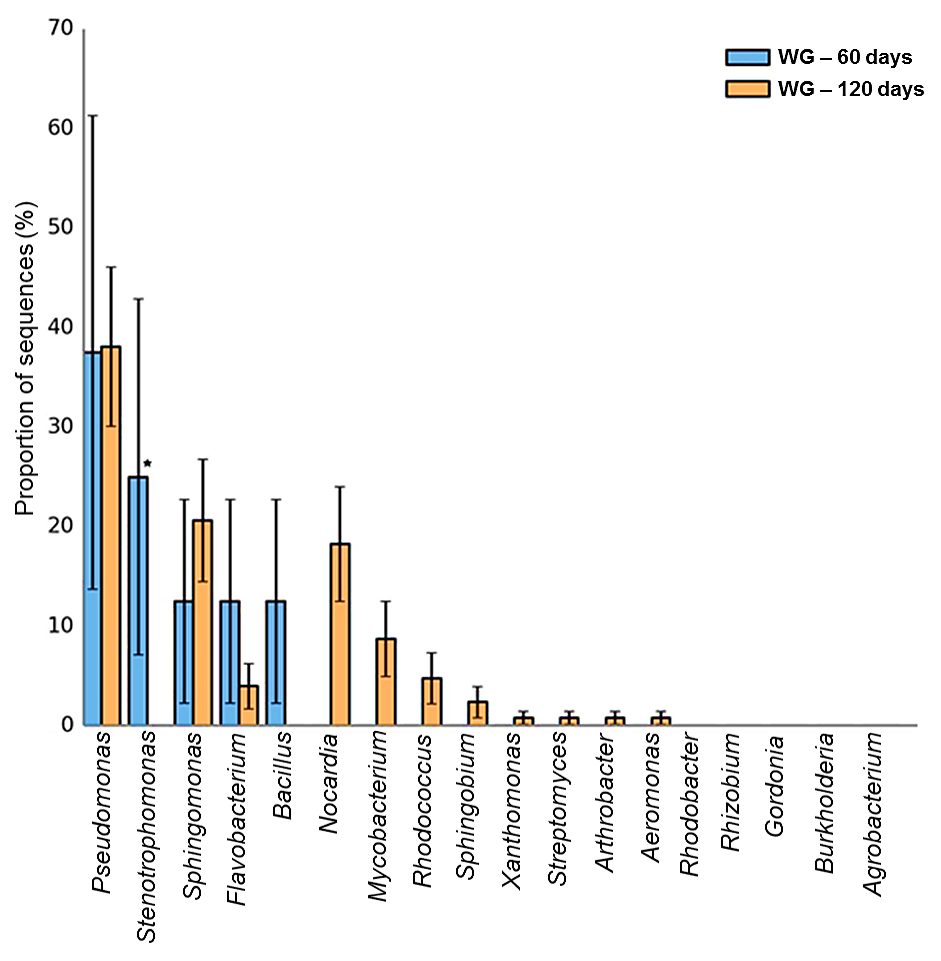
**

**Figure S11**. Variation in PAHs degrading bacterial genera of wallaby grass (WG) in 60^th^ and 120^th^ day rhizosphere soil sample. * - Statistical significance at *p* ≤ 0.05


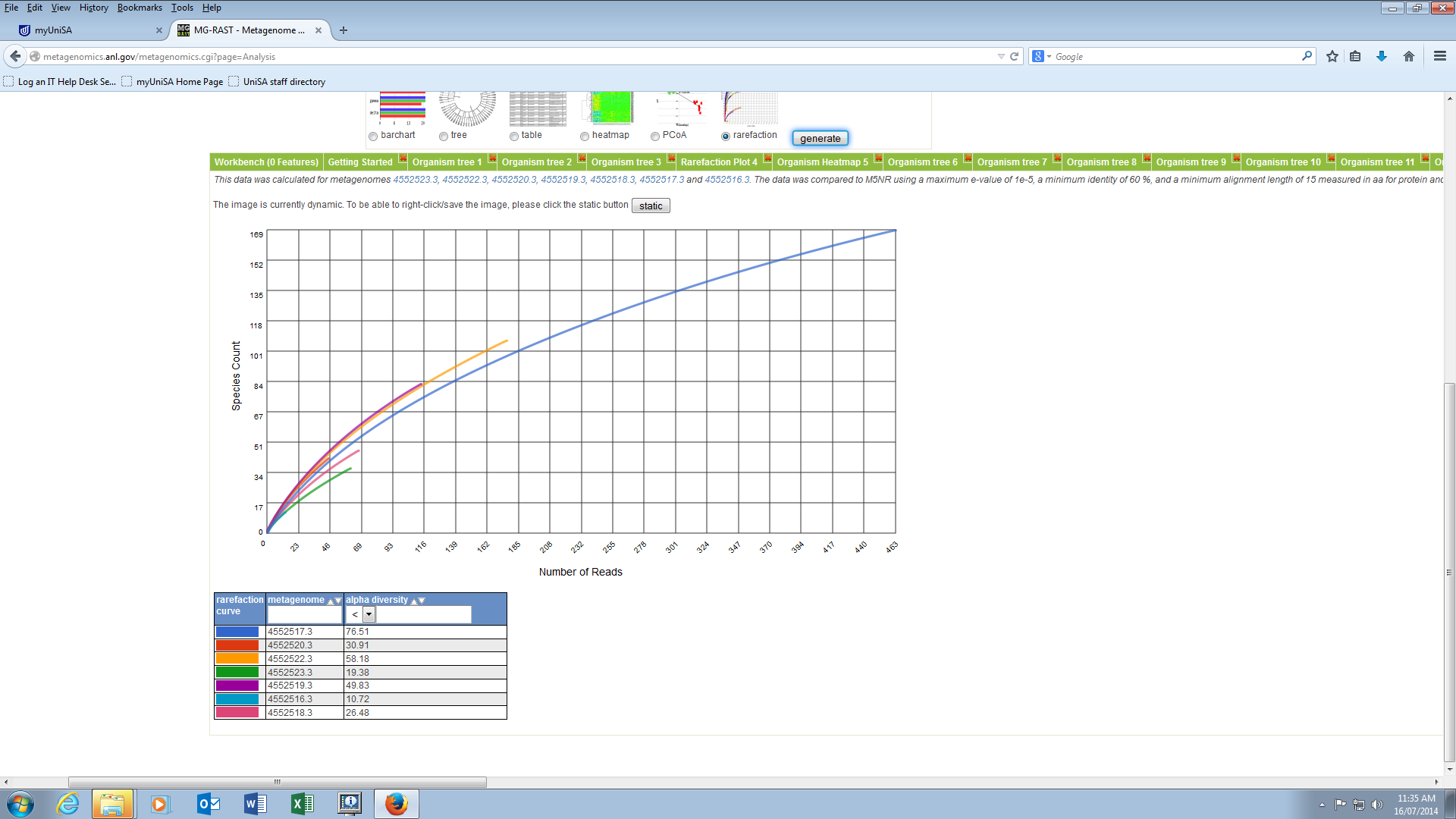

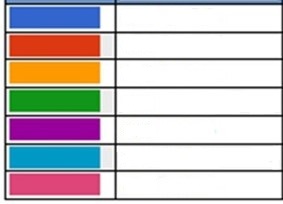


**MA**

**CP**

**SF**

**VV**

**SG**

**Unplanted**

**WG**

**Figure S12**. Rare fraction curve indicating microbial diversity among the samples after 60 days of growth. UP - unplanted, MA - maize, CP - cowpea, SF - sunflower, SG - Sudan grass, VV - vetiver and WG - wallaby grass

**
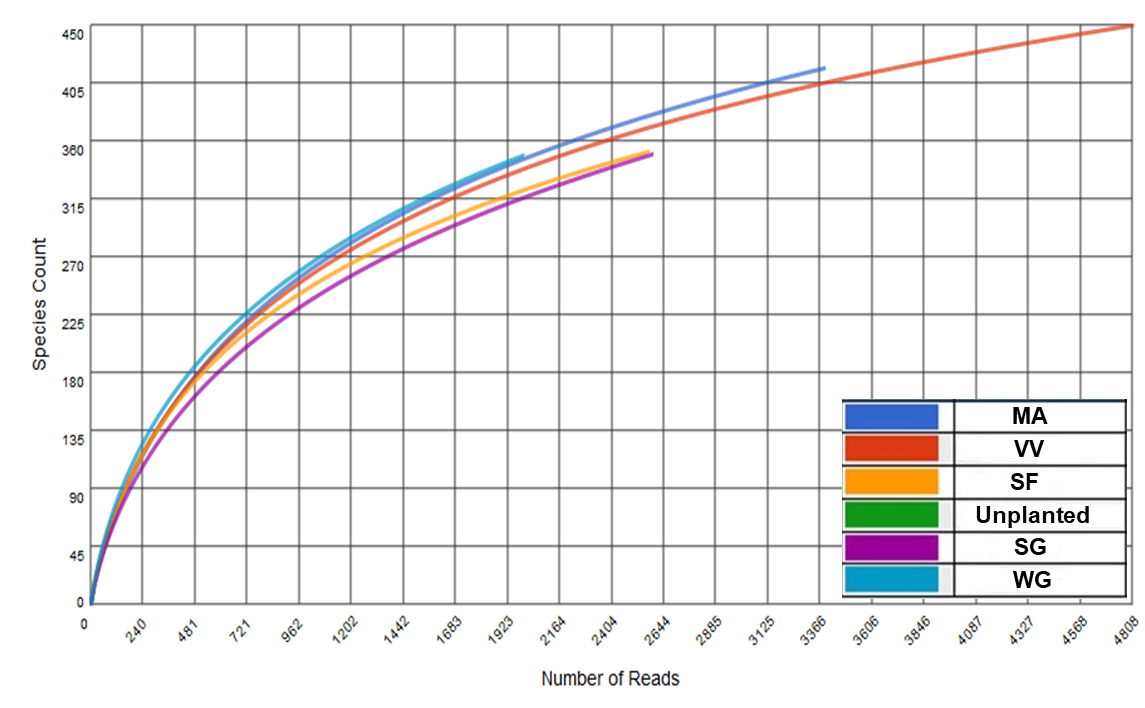
**

**Figure S13**. Rare fraction curve indicating microbial diversity among the samples after 120 days of growth. UP - unplanted, MA - maize, SF - sunflower, SG - Sudan grass, VV - vetiver and WG - wallaby grass.

**Table S1** Correlation co-efficient matrix between PAHs removal percentage and bacterial phylum abundance

| **Bacterial Phylum** | **% PAHs removal** |
| --- | --- |
| *Actinobacteria* | 0.87** |
| *Bacteroidetes* | - 0.01 |
| *Chlorobi* | 0.82** |
| *Cyanobacteria* | 0.79** |
| *Firmicutes* | 0.91** |
| *Proteobacteria* | 0.91** |
| *Verrucomicrobia* | 0.29 |

** Correlation is significant at the 0.01 level (2-tailed).

* Correlation is significant at the 0.05 level (2-tailed).

**Table S2** Correlation co-efficient matrix between PAHs removal percentage and bacterial genera abundance

| PAHs degrading bacterial genus | PAHs removal (%) – 60 days | | PAHs removal (%) – 120 days | |
| --- | --- | --- | --- | --- |
|  | C3 plants | C4 plants | C3 plants | C4 plants |
| *Acidovorax* | NA | NA | 0.458 | 0.689 |
| *Aeromonas* | NA | NA | NA | 0.684 |
| *Arthrobacter* | 0.957* | 0.966** | 0.947* | 0.966** |
| *Bacillus* | 0.781 | 0.321 | 1.000** | 0.945* |
| *Brevibacillus* | NA | NA | 0.573 | 0.802 |
| *Burkholderia* | NA | NA | NA | 0.467 |
| *Flavobacterium* | 0.98 | 0.493 | NA | 0.581 |
| *Microbacterium* | NA | NA | NA | 0.467 |
| *Micrococcus* | NA | NA | NA | 0.093 |
| *Mycobacterium* | NA | 0.493 | 0.573 | 0.986** |
| *Nitrosomonas* | NA | NA | 0.612 | 0.785 |
| *Nocardia* | 0.721 | 0.743 | 0.986** | 0.936** |
| *Pseudomonas* | 0.922** | 0.859 | 0.868 | 0.965** |
| *Rhizobium* | NA | NA | 0.942* | 0.981** |
| *Rhodococcus* | 0.721 | NA | 0.589 | 0.965* |
| *Sphingobium* | NA | NA | 0.212 | 0.845 |
| *Sphingomonas* | 0.988* | 0.996** | 0.591 | 0.998** |
| *Stenotrophomonas* | 0.984** | 0.997* | NA | 0.869 |
| *Streptomyces* | 0.958** | 0.973* | NA | 0.747 |
| *Xanthomonas* | NA | 0.987** | 0.834* | 0.984** |

** Correlation is significant at the 0.01 level (2-tailed).

* Correlation is significant at the 0.05 level (2-tailed).

**Table S3** Soil properties (Sivaram et al., 2018)

| **Soil properties** | **Concentration** |
| --- | --- |
| pH and EC | 8.5 and 322 µSm^-1^ |
| Soil moisture and water holding capacity | 9% and 52% |
| Dissolved organic carbon | 10 mg Kg^-1^ |
| Nitrogen and Carbon (%) | 0.04% and 2.6% |
| Napthalene | 8.0 mg Kg^-1^ |
| Acenaphthylene | 9.7 mg Kg^-1^ |
| Acenaphthene | 66.8 mg Kg^-1^ |
| Fluorene | 11.1 mg Kg^-1^ |
| Phenantherene | 45.3 mg Kg^-1^ |
| Anthracene | 21.4 mg Kg^-1^ |
| Fluoranthene | 56.4 mg Kg^-1^ |
| Pyrene | 105.9 mg Kg^-1^ |
| Benz[*a*]anthracene | 31.4 mg Kg^-1^ |
| Chrysene | 76.9 mg Kg^-1^ |
| Benzo[*b*]fluoranthene | 77.0 mg Kg^-1^ |
| Benzo[*k*]fluoranthene | 52.5 mg Kg^-1^ |
| Benzo[*a*]pyrene | 108.8 mg Kg^-1^ |
| Dibenz[*a,h*]anthracene | 92.9 mg Kg^-1^ |
| Benzo[*g,h,i*]perylene | 56.8 mg Kg^-1^ |
| Indeno [*123* *cd*]pyrene | 172.2 mg Kg^-1^ |

**Table S4** Soil Inorganics (Sivaram et al., 2018)

| **Inorganics** | **Concentration (mg Kg^-1^)** |
| --- | --- |
| Fluoride | 1.3 |
| Chloride | 28.4 |
| Bromide | < 0.5 |
| Nitrate | 9.8 |
| Sulphate | 70.6 |
| P | 126.3 |
| S | 214.3 |
| K | 3113.4 |
| Cr | 13.6 |
| Mn | 259.1 |
| Ni | 6.4 |
| Co | 11.2 |
| Cu | 15.6 |
| Zn | 34.2 |
| As | 17.9 |
| Cd | 0.1 |
| Pb | 22.0 |

**Table S5.** Linear mixed effects model for dehydrogenase activity (DHA) response on PAHs removal and experimental duration.

|  | **Value** | **SE** | ***t* - value** | ***p* - value** |
| --- | --- | --- | --- | --- |
| **Intercept** | -7.65 | 3.60 | -2.12 | 0.04 |
| **Days** | 13.62 | 4.46 | 3.05 | 0.004* |
| **Plant** | 1.363 | 0.92 | 1.47 | 0.21 |
| **Days: Plant** | -2.18 | 1.14 | -1.90 | 0.06* |

SE – Standard Error; * *p*-value is significant at the 0.05 level.

**Reference**

Sivaram, A. K. *et al.* Comparison of plants with C3 and C4 carbon fixation pathways for remediation of polycyclic aromatic hydrocarbon contaminated soils. *Scientific Reports* **8**, 2100 (2018).
